# Tetraspanins predict the prognosis and characterize the tumor immune microenvironment of glioblastoma

**DOI:** 10.1038/s41598-023-40425-w

**Published:** 2023-08-16

**Authors:** Yu-Chao Li, Yue Wu, Gang Chen, Li-Zhi Zhu, Xiu Luo, Qian-Qian Nie, Lu Zhang, Chang-Jing Zuo

**Affiliations:** 1 Department of Nuclear Medicine, The First Affiliated Hospital of Naval Medical University, Shanghai, China; 2 Department of Neurosurgery & Brain and Nerve Research Laboratory, The First Affiliated Hospital of Soochow University, Suzhou, China; 3 Department of Neurology & Brain and Nerve Research Laboratory, The First Affiliated Hospital of Soochow University, Suzhou, China

**Keywords:** Cancer, Computational biology and bioinformatics, Neuroscience

## Abstract

Glioblastoma (GBM) is the most aggressive and lethal primary brain tumor. Conventional treatments have not achieved breakthroughs in improving survival. Therefore, novel molecular targets and biomarkers need to be identified. As signal transduction docks on the cell membrane, tetraspanins (TSPANs) are associated with various tumors; however, research on their role in GBM remains extremely scarce. Gene expression and clinicopathological characteristic data were obtained from GEPIA, CGGA, HPA, cBioPortal, and GSCA databases to analyze the mRNA and protein expression levels, prognostic value, clinical relevance, mutation status, and targeted drug sensitivity of TSPANs in GBM. Gene set enrichment analysis (GSEA), Gene Ontology (GO), and the Kyoto Encyclopedia of Genes and Genomes (KEGG) analysis were used for biological process enrichment. Data from TCGA and TCIA were used to construct the tumor immune microenvironment landscape of TSPANs. Different R software algorithms were used to analyze the immune score, immune cell infiltration, and immune checkpoint correlation. Univariate and multivariate analyses were performed for TSPAN4, which had the most significant predictive prognostic value, and a nomogram model was constructed to predict individual outcomes. The expression and function of TSPAN4 were verified in vitro. TSPAN3/4/6/11/12/18/23/24/25/26/27/28/29/30/31expressions were significantly upregulated in GBM, and TSPAN3/4/6/11/18/24/25/26/29/30 were strongly correlated with prognosis. The expression of multiple TSPANs significantly correlated with 1p/19q co-deletion status, IDH mutation status, recurrence, age, and tumor grade. GSEA and GO analyses revealed the potential contribution of TSPANs in cell adhesion and migration. Immune correlation analysis revealed that TSPANs are related to the formation of the GBM tumor microenvironment (TME) and may influence immunotherapy outcomes. TSPAN4 is an independent prognostic factor and TSPAN4 knockdown has been demonstrated to strongly inhibit glioma cell proliferation, invasion, and migration in vitro. We comprehensively elaborated the prognostic value and potential role of differentially expressed TSPANs in GBM, including molecules that scientists have previously overlooked. This study provides a novel and comprehensive perspective on the pathological mechanisms of GBM and the future direction of individualized tumor immunotherapy, which may be a critical link between GBM malignant progression and TME remodeling.

## Introduction

Glioblastoma (GBM) is the most common primary malignant brain tumor, with many clinical complications, low patient quality of life, and an abysmal overall prognosis^1^. A comprehensive treatment plan based on surgical intervention, supplemented with temozolomide, postoperative chemotherapy, and radiotherapy was developed. However, after first-line treatment, almost all patients with GBM experience tumor recurrence and progression within 1 year^2^. In recent years, the development of immunotherapy has dramatically changed the treatment landscape for various solid tumors, bringing new hope to overcome cancer^3^. However, owing to the particular structure of the central nervous system and the unique immunosuppressive properties of glioma, no effective breakthrough in the application of immunotherapy in gliomas has been achieved^2^. Therefore, so far, no single treatment or drug has demonstrated a clear value in prolonging the overall survival (OS) of patients.

Genome-wide expression studies have reported GBM as a highly heterogeneous tumor with diverse epigenetic signatures, several gene amplifications, and rare mutations. Tailoring therapies for patients with unique molecular and genetic features is a promising strategy^4,5^. Therefore, identifying new molecular targets and biomarkers of GBM is particularly important for developing more effective therapeutic approaches and management options.

Tetraspanins (TSPANs) or transmembrane 4 superfamily proteins, contain four highly conserved transmembrane domains structurally that are highly homologous across species. Members of this family are present in almost all eukaryotic cell types and tissues, with 33 isoforms identified in mice and humans^6^.TSPANs are often referred to as molecular organizers of the plasma membrane and function as scaffolds that anchor multiple proteins to a region of the cell membrane, forming functional units called TSPAN-enriched microstructural domains (TEMs) that transduce signals. TEMs typically contain several TSPANs, integrins, adhesion receptors, immunoglobulin superfamily proteins, extracellular enzymes, and cytokine receptors^7^.

Previous studies have reported that the TSPAN receptor family is involved in a surprisingly large number of biological processes and plays essential roles in cell signaling, cell adhesion/migration, vascular morphogenesis and remodeling, immune system regulation, and inflammation^6–8^. In particular, TSPANs are also actively involved in various pathological conditions, including viral infections, diabetes, hepatitis, and tumor metastasis/progression^9–12^. However, elucidation of the overall expression profile of TSPANs and the relevance of their potential role and clinical significance in the malignant progression of GBM and the formation of an immunosuppressive microenvironment remain very limited.

Based on integrated machine learning methods to analyze data from multiple large public databases, this study provides the first systematic assessment of the differential expression, prognostic value, and immune infiltration correlation of TSPANs in GBM and makes a preliminary in vitro experimental validation of the member with the most significant predictive prognostic value. Our data will provide new insights into the basic theory and clinical application of GBM, and may help predict GBM risk, treatment outcome, and prognosis, and even provide potential immunotherapeutic options.

## Material and methods

### Gene expression profiling interactive analysis (GEPIA)

Gene Expression Profiling Interactive Analysis (GEPIA) web-based bioinformatics tool (http://gepia.cancer-pku.cn/) is a server for cancer and normal gene expression profiling and interactive analyses^13^. It was used in this study to perform a pan-cancer analysis of tetraspanins and their differential expression analysis in GBM. Significance was set at p < 0.05.

### Chinese glioma genome atlas (CGGA) database

Tetraspanins RNA-seq expression, matched survival probabilities, and clinicopathological data were obtained from the Chinese Glioma Genome Atlas (CGGA) database^14^ (http://www.cgga.org.cn) for prognostic and clinical correlation analyses. Univariate and multivariate analyses were performed using the R package Library (survival) package Version 3.3.1. Further nomograms, including glioma-related clinical parameters and independent prognostic factors, were constructed using the following R packages: library (survival) package Version 3.3.1, library(regplot) package Version 1.1, and library(rms) package Version 6.3.0.

### Protein level analysis

Immunohistochemical data from The Human Protein Atlas (HPA: https://www.proteinatlas.org/) database were downloaded to explore the differences in TSPAN protein expression between GBM and normal tissues. The interactions between TSPAN family proteins were analyzed using the GluGo plugin of the Cytoscape software, and the enriched biological process networks were visualized. We only report pathways with p < 0.05.

### TCGA and TCIA databases

The transcriptome data of glioma in The Cancer Genome Atlas (TCGA) database (https://portal.gdc.cancer.gov/) was downloaded, and Gene Set Enrichment Analyses (GSEA) of differentially expressed TSPANs were performed using the packages “org.Hs.eg.db” (package Version 3.15.0), “clusterProfiler” (package Version 4.4.4) and “enrichplot” (package version 1.16.1). Optional data sets were “c5. Go. V7.4. Symbols. GMT”, “c2. Cp. Kegg. V7.4. Symbols. GMT". Then we used the R packages “estimate” (package Version 1.0.13), “reshape2” (package Version 1.4.4) and “ggpubr” (package Version 0.4.0) to draw violin maps for immune microenvironment analysis. R packages “limma” (package Version 3.52.2), “reshape2” (package Version 1.4.4), “ggpubr” (package Version 0.4.0), “vioplot” (package Version 0.3.7) and “ggExtra” (package Version 0.10.0) were used for differential and correlation analysis of immune cells, and box plots and Lollipop plots were drawn for visualization. For further immune checkpoint correlation analysis, the “limma” (package Version 3.52.2), “reshape2” (package Version 1.4.4), "ggplot2"(package Version 3.3.6), “ggpubr” (package Version 0.4.0), “corrplot” (package Version 0.92) packages were used, and both circle plots and correlation heatmaps were drawn simultaneously. The Cancer Imaging Archive (TCIA, https://cancerimagingarchive.net/) and TCGA data were used to analyze the efficacy of immunotherapy in both high- and low- expression TSPANs using the R packages limma (package Version 3.52.2) and ggpubr (package Version 0.4.0). R package survival (package Version 3.3.1), survminer (package Version 0.4.9), and timeROC (package Version 0.4) were used to plot receiver operating characteristic (ROC) curves for predicting OS at 1, 3, and 5 years in patients with glioma, and the area under the curve (AUC) representing the accuracy of diagnostic techniques was calculated. Statistical significance was set at p < 0.05.

### Funrich bioinformatics tool

FunRich (http://www.funrich.org) is a user-friendly bioinformatics tool that can be used to perform enrichment analysis of omics datasets^15^. We used FunRich to analyze the intersecting genes of the 33 tetraspanins and plotted the results as a network graph.

### DAVID knowledgebase

To investigate the signaling pathways and potential functions associated with the TSPAN family and their intersecting genes, we used the DAVID Knowledgebase v2022q3 (https://david.ncifcrf.gov/) performed Kyoto Encyclopedia of Genes and Genomes (KEGG)^16–18^ and Gene Ontology (GO) analyses, and the top ten enrichment items were drawn into a bubble map. A p-value < 0.05 was considered statistically significant.

### Mutational profile and drug sensitivity analysis of TSPANs

The cBioPortal platform (http://www.cbioportal.org/) was used to analyze mutations in the TSPAN family genes in gliomas. Data from the Gene Set Cancer Analysis (GSCA) (http://bioinfo.life.hust.edu.cn/GSCA/) database were used to analyze the relevant signaling pathways and drug sensitivity of the TSPAN gene family. All drugs were approved by the Food and Drug Administration or passed clinical trials.

### Cell culture and transfection

Three glioma cell lines, U251, U87, and T98G, were obtained from the Cell Bank of the Chinese Academy of Sciences (Shanghai, China). Glioma cells were cultured in F12 medium (Corning, NY, USA) mixed with 10% foetal bovine serum (Gibco, MA, USA) in an incubator at 37 °C and 5% CO_2_ and used as an in vitro model for analysis. Small interfering RNAs targeting TSPAN4 were designed and synthesized by GenePharma (Shanghai, China) to knock down the expression of TSPAN4. Transfection was performed according to the manufacturer’s instructions, and the cells were inoculated into six-well plates and transfected when they reached 70% confluence. To obtain the siRNA dilution solution, 100 pmol siRNA was diluted with 250 µl Opti-MEM medium, added with 5 µl lipofectamine 2000 to 250 µl Opti-MEM medium, and incubated for 5 min at room temperature. The liquid obtained from the above two steps was mixed and incubated for 20 min at room temperature to obtain a transfection solution (final siRNA concentration of 33 nM) and added to 6-well plates. Subsequently, the medium was changed after 6 h of transfection.

The siRNA target sequences are as follows: TSPAN4-Homo-400; sense (5′-3′): GUGCCAUCAAGGAGAACAATT; and antisense(5′-3′): UUGUUCUCCUUGAUGGCACTT.

### RNA isolation and real-time quantitative polymerase chain reaction

Total cellular RNA was extracted using the TRIzol reagent (Invitrogen, Carlsbad, CA, USA). A one- drop OD-1000 + 140 spectrophotometer was used to determine total RNA concentration. cDNA was synthesized using a RevertAid First Strand cDNA Synthesis Kit (#K1621, Thermo Scientific, USA). qRT-PCR was performed using SYBR™Green PCR Master Mix (#43,091,055; ThermoFisher Scientific, USA) according to the manufacturer’s instructions. mRNA relative expression was normalized by glyceraldehyde 3-phosphate dehydrogenase and calculated by relative quantification (2-ΔΔCt). Each sample was repeated three times. Primers were purchased from RIBOBIO (China), and the primer sequences are as follows: genDETECTTM h-TSPAN4_qPCR_161bp_F1: positive-sense strand (5′-3′) CGGAACCCTGTTTCTGGAAG; GQP00070788 genDETECTTM h-TSPAN4_qPCR_161bp_R1: positive-sense strand (5′-3′) CGTGTGAACACTGCTTTGGAGA.

### Western blot analysis

Cell lysis buffer for western blotting (Cat: P0013; Beyotime, Shanghai, China) was used to extract total protein from the pretreated cells. The protein concentration of whole-cell lysates was determined using a bicinchoninic acid protein assay kit (Beyotime, Shanghai, China). Protein concentrations were leveled to 3 mg/mL, and the loading volume was 10 μL. Protein samples were separated by electrophoresis on standard sodium dodecyl sulfate–polyacrylamide gel electrophoresis gels and transferred to 0.2 mm polyvinylidene difluoride membranes (Millipore). The membranes were blocked with 5% bovine serum albumin for 1 h at room temperature and then incubated with the primary antibody overnight at 4 °C, followed by 1 h of incubation at room temperature using a matching secondary antibody. Antibody source: anti-E-cadherin (CST#94386; dilution for western blot, 1: 1000), anti-N-cadherin (CST#13116; dilution for western blot, 1:1000), anti-Vimentin (CST#46173; dilution for western blot, 1:1000), anti-MMP9(CST#15749; dilution for western blot, 1:1000), anti-MMP2(CST#40994; dilution for western blot, 1:1000), and anti-β-Tubulin (CST#2146; dilution for western blot, 1:1000). Finally, images were acquired using a CLINX imaging system and quantified using the ImageJ software.

### Transwell migration assay

The transwell assay was used to detect the migration and invasion abilities of U-87, T98G, and U-251 cells. Briefly, cells were seeded in Transwell chambers (Corning, USA) pre-coated with or without Matrigel (Corning, USA). Serum-free medium was injected into the upper chamber, and Dulbecco’s modified eagle medium was added to the lower chamber. After incubation at 37 °C for 24 h, the cells were fixed with 4% paraformaldehyde and stained with 0.1% crystal violet. Finally, the cells were counted under a microscope.

### Colony formation assay

The colony formation assay is an effective method to test the proliferative ability of cultured cells by calculating the colony-forming efficiency. Five hundred cells were seeded in six-well plates and cultured in complete medium for 1 week. When cell colonies were visible to the naked eye, the culture medium was discarded, and 2 ml of methanol was added to each well and fixed for 30 min. Methanol was discarded, and 2 ml 0.1% crystal violet was added to each well for staining for 3 min, after which the crystal violet was rinsed. Finally, the cell colonies were imaged and counted.

### Wound healing assays

The cells were seeded in six-well plates containing serum and cultured for 1–2 days. When the cell density reached 90%, a 200µl sterile pipette tip was used to scratch the monolayer. Dead cells were removed by rinsing twice with phosphate buffer solution. The cells were cultured in serum-free medium for 0, 24, and 48 h. Lesion edge images were acquired using an inverted microscope. The wound healing capacity was calculated from the acquired images.

### Statistical analysis

Bioinformatic analyses and R packages were performed using the R software, version 4.2.0. A Cox regression analysis was performed to calculate hazard ratios to analyze the association between TSPAN4 expression and survival. For molecular biology experiments, GraphPad Prism 8 was used for statistical analysis, and graphs were generated. To detect the differences between the data of the intervention and control groups, the data of the two groups were independent of each other, and the outcome variables were continuous variables, conforming to a normal distribution with equal variances, using two independent samples *t* test; to detect the expression between different cell lines, the data of each group were independent of each other, and the outcome variables were continuous variables, conforming to normal distribution with equal variances, using a one-way analysis of variance. All molecular biology experiments were repeated at least three times. *p < 0.05, **p < 0.01, and ***p < 0.001 were considered significant.

## Results

### Transcriptional levels of different TSPANs in patients with GBM

**Figure 1:**
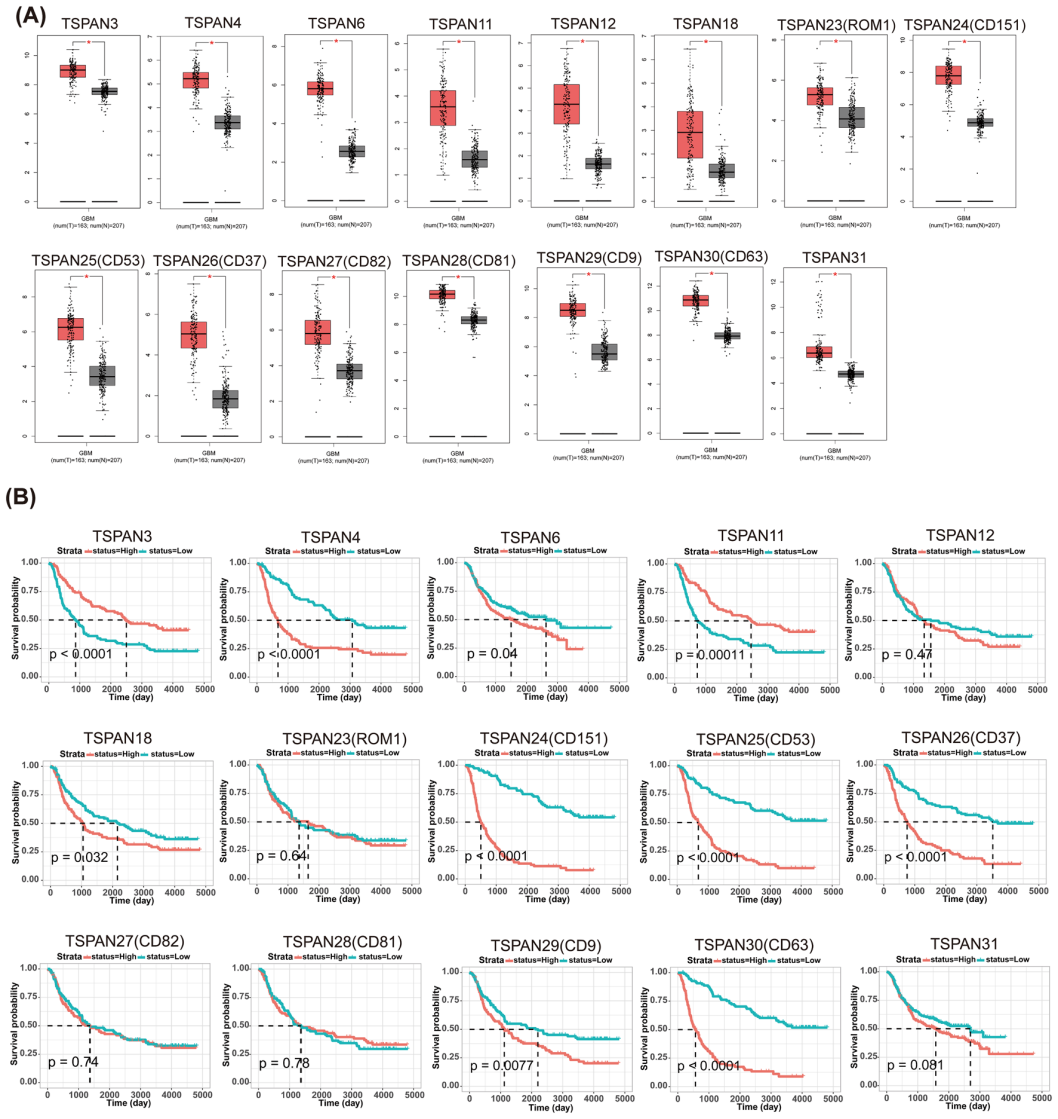
Expression profile and prognostic value of TSPANs in patients with GBM. (**A**) TSPAN3/4/6/11/12/18/23/24/25/26/27/28/29/30/31 were significantly upregulated in GBM compared to the normal group, *p < 0.05. (**B**) The overall survival of patients in different TSPANs low- and high- expression groups were compared by Kaplan–Meier analysis based on the CGGA database. The red curve represents patients with increased expression of TSPANs, and the blue curve represents patients with low TSPANs. Significance was set at p < 0.05.

Based on the GEPIA database, we compared the transcript levels of TSPANs in tumor and normal tissues. Pan-cancer analysis revealed that the vast majority of TSPAN family members were differentially expressed in multiple tumor types, including prostate adenocarcinoma, rectal adenocarcinoma, skin cutaneous melanoma, testicular germ cell tumors, cholangio carcinoma, colon adenocarcinoma, lower-grade brain glioma, pancreatic adenocarcinoma and GBM (see Additional Files 1). In GBM, the mRNA expression levels of TSPAN3/4/6/11/12/18/23/24/25/26/27/28/29/30/31 were significantly upregulated compared to those in normal tissues (Fig. 1A, p < 0.05), whereas the transcript levels of the remaining TSPAN family molecules were not significantly different.

### Prognostic values of TSPANs in patients with glioma

Next, we used the CGGA online database to assess the prognostic value of the above 15 significantly upregulated TSPANs mRNA in patients with glioma and plotted Kaplan–Meier survival curves. As presented in Fig. 1B, the expression levels of TSPAN3/4/6/11/18/24/25/26/29/30 were significantly correlated with the OS of patients with glioma (p < 0.05). Specifically, the higher the TSPAN3/11 level, the greater the probability of survival in patients with glioma. By contrast, the survival advantage of patients in the high TSPAN4/6/18/24/25/26/29/30 expression group was significantly lower than that of the patients in the low expression group. Thus, TSPAN3/11 may play a protective prognostic role, whereas TSPAN4/6/18/24/25/26/29/30 are poor prognostic factors, demonstrating that they may be potential biomarkers for glioma and serve as predictive signals for different prognoses. The expression levels of the remaining TSPAN family members had no significant prognostic value.

### Potential clinical value of TSPANs in patients with glioma

To explore the relationship between the 15 differentially expressed TSPAN family members and glioma-related clinical features, we analyzed the CGGA database. As presented in Table 1, the mRNA expression of TSPAN3/4/6/11/12/18/24/25/26/29/30 was significantly correlated with the individual cancer grades (p < 0.05). 1p/19q co-deletion and isocitrate dehydrogenase 1 (IDH1) or isocitrate dehydrogenase 2 (IDH2) gene mutation were used as diagnostic markers for adult diffuse gliomas by the World Health Organization (WHO) in 2016^19^. According to the results of our analysis, the transcript levels of TSPAN3/4/11/12/18/24/25/26/30 were significantly correlated with IDH mutation status (p < 0.05). Meanwhile, the expression of TSPAN3/4/11/24/25/26/28/29/30 was associated considerably with the 1p/19q co-deletion status (p < 0.05). Moreover, TSPAN3/6/18/28/30 expression was significantly different between primary and recurrent tumors (p < 0.0). We then extracted data on the correlation of TSPANs with sex and age of patients with GBM. Only TSPAN30 demonstrated significant differences between sexes (p < 0.05). TSPAN3/4/11/12/24/25/26/30 demonstrated a significantly different expression in patients aged < 42 years compared with those aged > 42 years (p < 0.05).

**Table 1:** Correlation between the differentially expressed genes of the TSPAN family and clinical features of glioma.

The clinical features of glioma	The differentially expressed genes of TSPAN family	TSPAN3	TSPAN4	TSPAN6	TSPAN11	TSPAN12	TSPAN18	TSPAN23 (ROM1)	TSPAN24 (CD151)	TSPAN25 (CD53)	TSPAN26 (CD37)	TSPAN27 (CD82)	TSPAN28 (CD81)	TSPAN29 (CD9)	TSPAN30 (CD63)	TSPAN31
Grade	p value	9E-10	5.90E-20	0.0077	3.1E-07	0.035	0.0028	0.92	9.6E-34	1.3E-08	3.6E-08	0.12	0.06	0.016	4.5E-34	0.055
IDH mutation status		1.7E-18	1.5E-19	0.52	1E-17	5.5E-07	0.00074	0.12	8.9E-32	6.6E-11	2.1E-11	0.83	0.13	0.071	4E-31	0.99
1q/19q co-deletion status		4.5E-07	0.001	0.16	7.8E-10	0.14	0.51	0.93	3.1E-24	3E-18	9.9E-15	0.65	0.00078	0.017	1.6E-18	0.31
Sex		0.31	0.16	0.77	0.84	0.63	0.39	0.22	0.11	0.32	0.99	0.68	0.11	0.12	0.036	0.39
Age		2.9E-05	1.8E-06	0.57	2.6E-05	0.0025	0.76	0.86	6E-10	0.0053	0.0019	0.69	0.16	0.1	1.9E-08	0.73
Progression status		0.029	0.26	0.023	0.2	0.5	0.021	0.75	0.28	0.6	0.5	0.15	0.0042	0.18	0.066	0.62

Thus, TSPANs may serve as biomarkers for testing the severity and heterogeneity of GBM, and have a potential role in predicting tumor recurrence, clinical diagnosis, and treatment.

### Drug sensitivity analysis of TSPANs in patients with GBM

Using the GSCALite online tool, we evaluated the role of TSPANs levels in sensitivity to small molecule targeted antineoplastic drugs. A positive Spearman correlation indicated that high TSPAN expression was resistant to the drug. The results are presented in Additional File 2. The expression of TSPAN1/4/6/15/17/24/29/30 was positively correlated with most drugs, whereas that of TSPAN25/26/32 was mostly negatively correlated. These results indicate that the TSPAN family is closely related to drug resistance in antitumor and immunotherapy, suggesting that TSPANs may be potential targets for GBM therapy.

### Levels of different TSPANs in glioma tissues

Next, we used the HPA database to obtain immunohistochemical information on TSPANs in normal and glioma tissues. As presented in Additional File 3, TSPAN1/6/9/12/14/16/17/19/20/ 28/30/31 were undetectable in normal tissues but indicated low or moderate expression in GBM tissues. In particular, TSPAN24, which was not detected in normal tissues, was significantly upregulated in tumor tissues, showing high expression. TSPAN2/13/25/29 showed low expression in normal tissues but medium to high expression in tumor tissues. TSPAN3 is moderately expressed in normal tissues but highly expressed in tumor tissues. Overall, our results suggest that ectopic expression of the 18 members mentioned above of the TSPAN family is an available feature in patients with GBM and may help in the diagnosis of GBM.

### Biological function enrichment analyses of TSPANs

**Figure 2:**
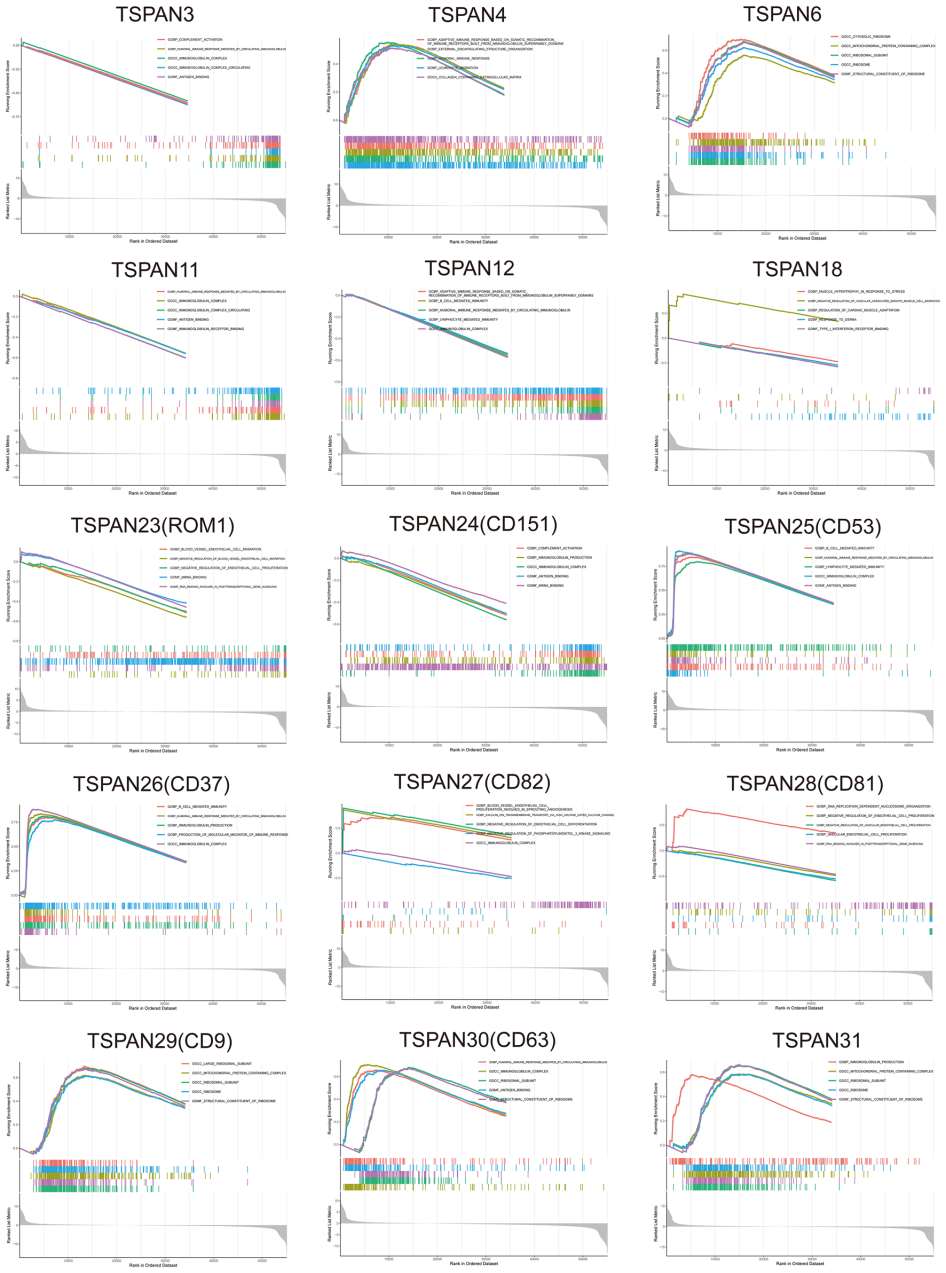
Gene set enrichment analysis of TSPANs in patients with glioblastomas. Gene set enrichment analysis (GSEA) was conducted to investigate the biological processes involved in TSPANs in glioma. The results revealed that TSPANs were highly correlated with immune response.

To investigate the possible molecular mechanisms of the 15 differentially expressed TSPAN gene signatures in GBM and to elucidate the related biological functions and pathways, we downloaded the transcriptome data of GBM from TCGA database for GSEA enrichment analysis. The selected datasets were “c5.go.v7.4. symbols.gmt” (Fig. 2), “c2.cp.kegg.v7.4.symbols.gmt” (Additional File 4), p < 0.05, and the top five enrichments for each subtype were visualized. The results indicated that TSPANs were mainly enriched in immunoglobulin complex production, humoral immunity, cellular immunity, vascular endothelial cell proliferation, cytokine-cytokine receptor interactions, chemokine signaling pathways, cell adhesion molecules cams, natural killer cell-mediated cytotoxicity, antigen processing, presentation, and other related pathways.

**Figure 3:**
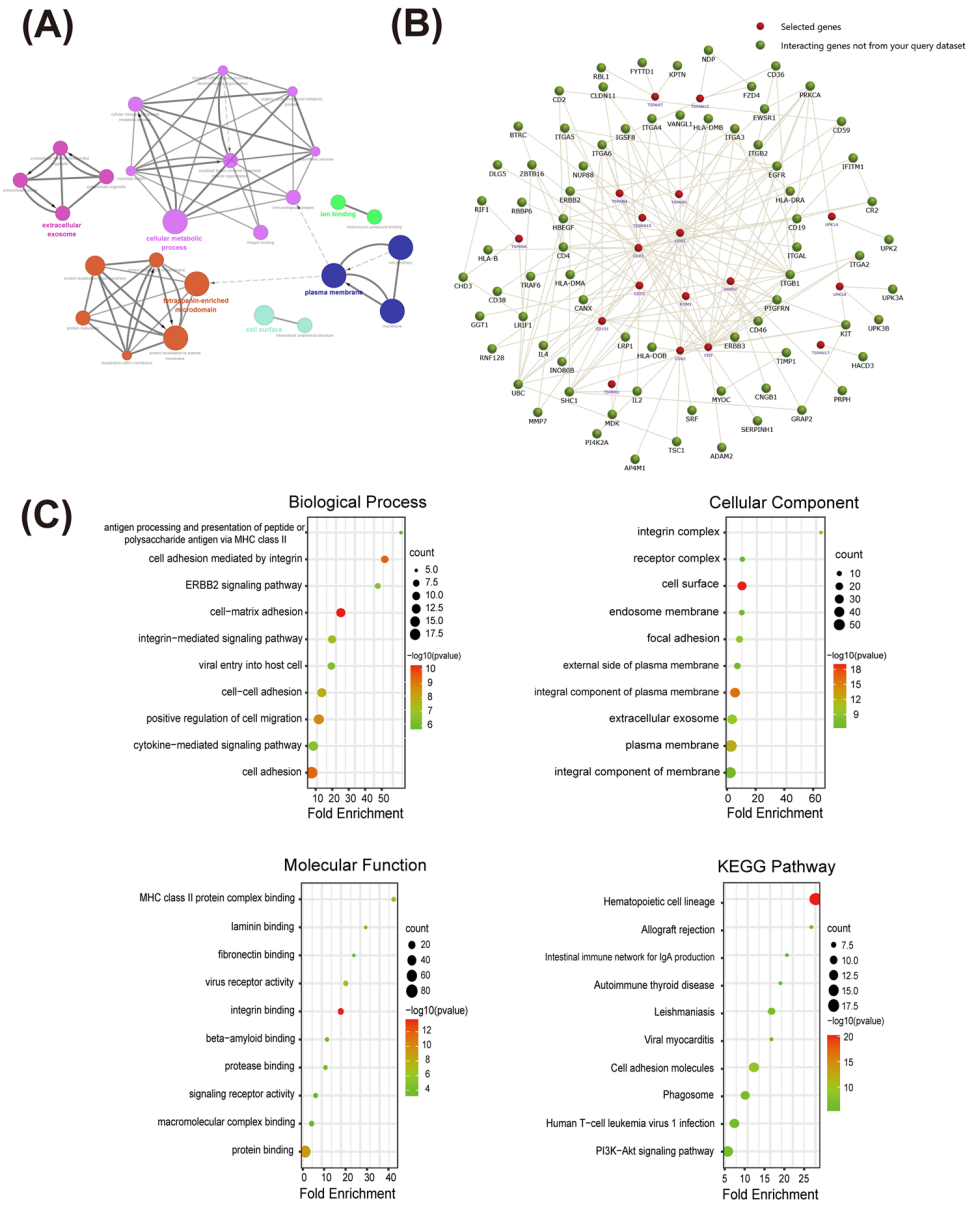
Functional enrichment and intersection gene analysis of TSPANs. (**A**) Functional enrichment analysis of the TSPAN family using ClueGO and mapping of interaction networks. Multiple-colored dots indicate that TSPANs are involved in various biological processes. (**B**) Intersecting genes of TSPANs were analyzed using the FunRich software, and the network was plotted. Red dots represent TSPAN family genes (TSPANS not enriched in intersection genes are not shown), and green dots represent enriched intersection genes. (**C**) GO and KEGG functional enrichment analyses of TSPANs and their intersecting genes based on the DAVID platform. The dot size represents the gene count and the bubble color represents the q-value.

Additionally, we visualized the functional annotation analysis network of the TSPANs using the GLUEGO plugin of Cytoscape. As presented in Fig. 3A, TSPANs were mainly associated with extracellular exosomes, cellular metabolic processes, tetraspanin-enriched microdomains, plasma membranes, cell surfaces, and ion binding.

These results suggest that tetraspanins play an indispensable role in signal transduction and the immune response and may be closely related to the formation of the tumor microenvironment (TME) in GBM.

**Figure 4:**
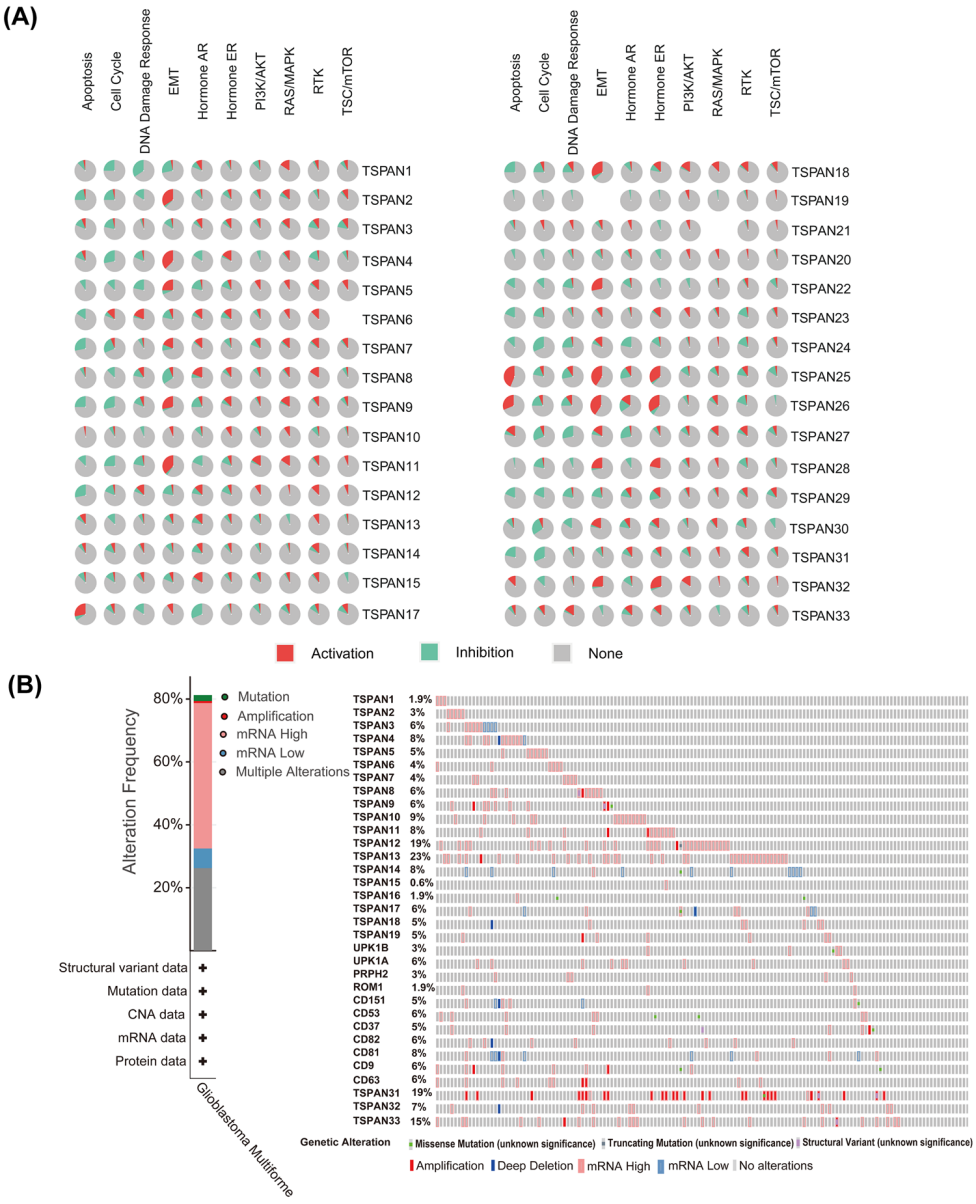
Analysis of signaling pathway correlations and mutation profiles of the TSPAN family. (**A**) Querying the TSPANs family for related molecular signaling pathways involved in glioblastoma (GBM) based on the Gene Set Cancer Analysis database. Red represents activation of the pathway; green represents inhibition of the pathway. (**B**) Analysis of TSPAN family mutations in GBM based on the cBiPortal database.

Next, we explored the roles of TSPANs and their functionally intersecting genes. The 91 genes intersecting with TSPANs at the gene level were obtained using Funrich enrichment (Fig. 3B) and uploaded to the DAVID database for GO and KEGG enrichment analysis (Fig. 3C). The results revealed that TSPANs and their neighboring genes were mainly enriched in biological processes related to cell adhesion, migration, and signal transduction. In particular, the ERBB2 signaling pathway, which generally predicts high malignancy and poor prognosis in tumor cells^20^, has also been closely linked to these genes. These genes are involved in cellular components such as the cell surface, an integral component of the plasma membrane, and the plasma membrane. The molecular functions of these genes were mainly to regulate signal transduction by binding to integrins and proteins. The main KEGG pathway enriched in these genes was the hematopoietic cell lineage. A further pathway activity study (Fig. 4A) revealed that TSPAN2/4/5/9/11/18/22/25/26/28/32 were significantly associated with the activation of the epithelial-mesenchymal transition (EMT) pathway, a critical pathway for cancer cell metastasis^21^. TSPAN1/4/9/11/24/29/30/31 is associated with inhibition of the cell cycle pathway.

### Genetic mutations of TSPANs in GBM

To further explore the possible regulatory mechanisms of the TSPAN family members in the development and progression of GBM, we analyzed the mutation data of TSPANs using the cBioPortal database. As presented in Fig. 4B, TSPANs expression was altered in 160 of the 155 patients with GBM. The mutation frequencies of the 15 TSPAN family members with prognostic significance were TSPAN3 (6%), TSPAN4 (8%), TSPAN6 (4%), TSPAN11 (8%), TSPAN12 (19%), TSPAN18 (5%), TSPAN23 (1.9%), TSPAN24 (5%), TSPAN25 (6%), TSPAN26 (5%), TSPAN27 (6%), TSPAN28 (8%), TSPAN29 (6%), TSPAN30 (6%), and TSPAN31 (19%). Mutation patterns in TSPANs include missense mutations, truncating mutations, structural variants, amplifications, and deep deletions.

### TME landscape of differentially expressed TSPANs

**Figure 5:**
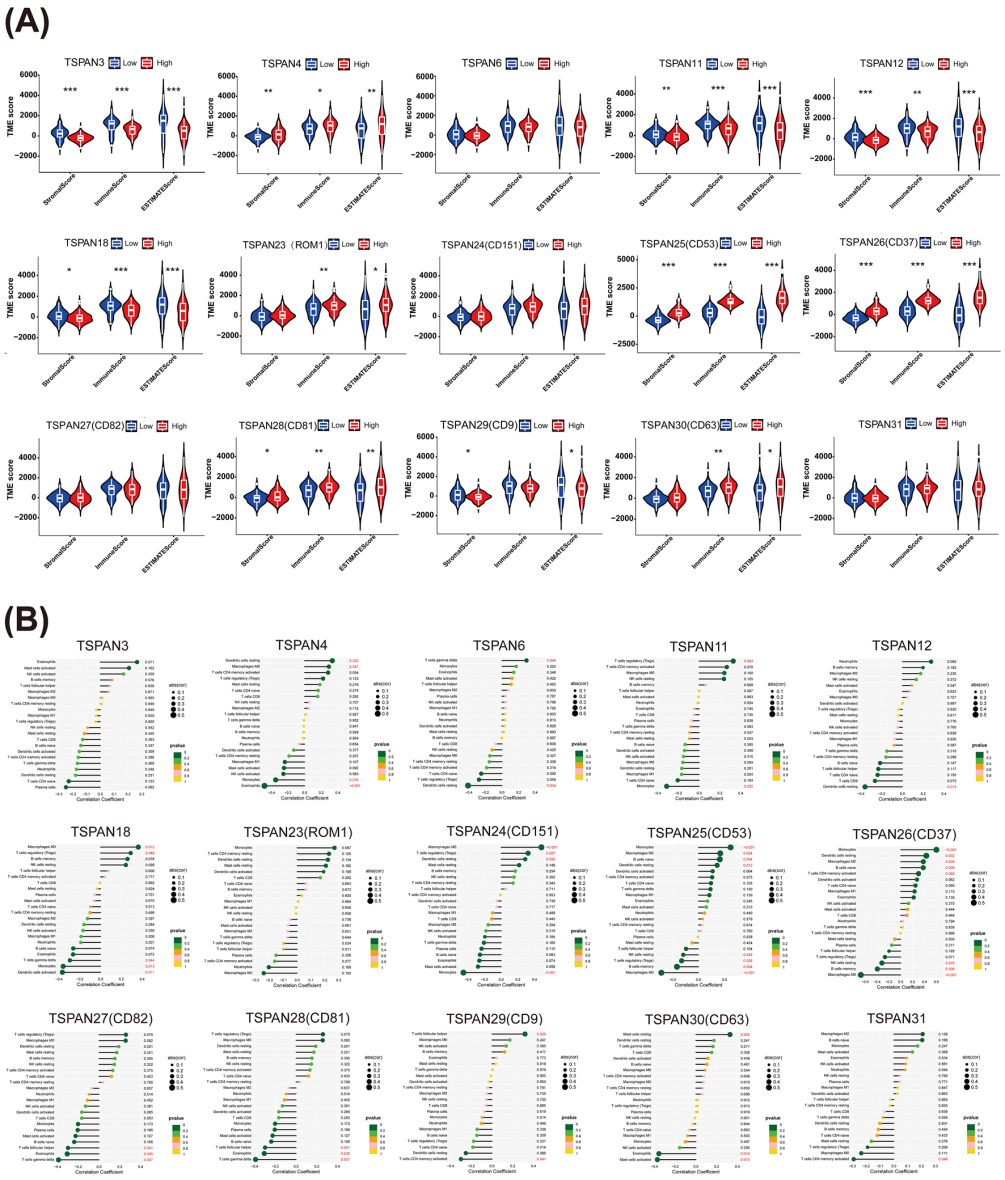
Immune/stromal score and immune cell correlation analysis of TSPANs. (**A**) TSPANs were scored by tumor microenvironment based on glioblastoma data in the TCGA database. Immune score, Stromal score and ESTIMATE score represent the relative proportions of immune and stromal components and their sum, respectively. The higher the score, the more significant the ratio of the corresponding element in the TME. Red represents patients with high TSPAN expression, and blue represents patients with low TSPAN expression. *p < 0.05, **p < 0.01, ***p < 0.001. (**B**) Lollipop plots of the relationship between TSPAN expression and different tumor immune lymphocytes. Significance was set at p < 0.05.

Compared with other solid tumors, the unique immune microenvironment of GBM poses a severe obstacle to the development of precise targeting and immune therapy^22^. Therefore, we performed an in-depth analysis of the TME and tumor-infiltrating immune cells. Studies have demonstrated that a high ratio of immune and stromal components in the TME may lead to worse prognosis. Therefore, to assess the proportion of immune and stromal components in the TME, we compared the stromal and immune scores between the low and high expression groups of TSPANs using TCGA database, which reflects the infiltration level of stromal and immune cells in the TME, respectively. The estimated score was the sum of the two ratings. The violin plot revealed that the three scores were generally higher for the low-expression TSPAN3/11/12/18/23/29, in contrast to TSPAN4/25/26/28/30, which was higher in the high-expression group (Fig. 5A, p < 0.05).

Next, we investigated the proportion of 22 immune cell subtypes in GBM samples from different high- and low-TSPAN groups. As demonstrated in Fig. 5B, the cell abundance of resting dendritic cells, M0 and M2 macrophages, gamma-delta (γδ) T cells, Tregs, naive CD4 T cells, follicular helper T cells (Tfh), monocytes, Naive B cells, memory b cells, resting mast cells, eosinophils, and resting NK cells were affected by TSPAN expression, demonstrating that the TSPAN family is closely associated with the immune infiltration of GBM.

**Figure 6:**
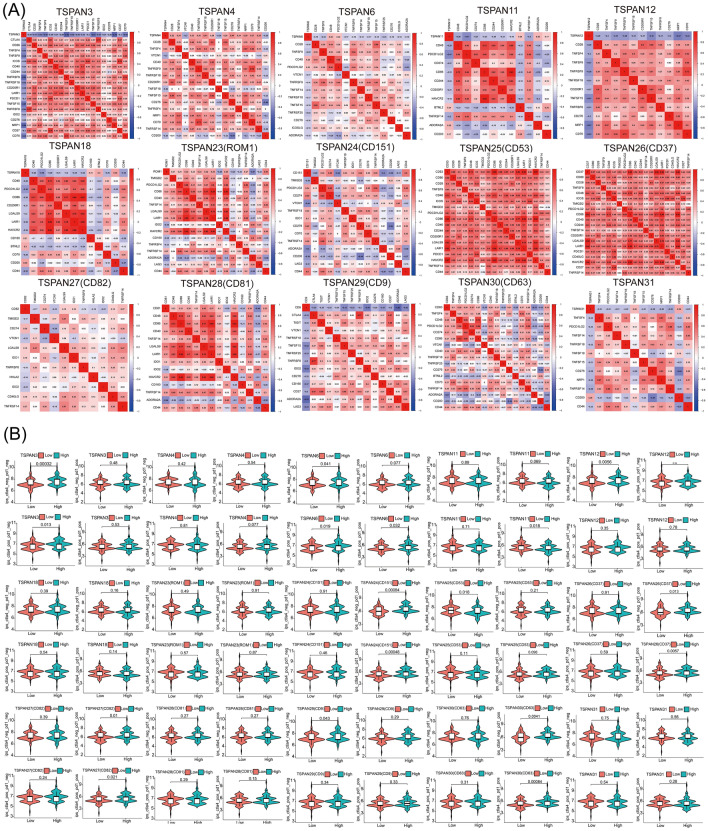
Analysis of the correlation between TSPANs and immune checkpoints and immunotherapy. (**A**) Heat map of the correlation between TSPANs and immune checkpoints. Red color blocks represent positive correlations; blue color blocks represent negative correlations. (**B**) Efficacy analysis of immunotherapy for TSPANs with different expression levels based on TCGA and TCIA databases. Those above the median of GBM-related transcriptome data were considered the high expression group, while those below the median were considered the low expression group. The high TSPAN3 expression subgroup was effective for CTLA4 + /PD1 − immunotherapy. The high TSPAN6 expression subgroup had a good response to CTLA4 + /PD1 − , CTLA4 + /PD1 + immunotherapy. The TSPAN11 low expression subgroup was effective for CTLA4 + /PD1 + immunotherapy. The subgroup with high TSPAN24/26/27/30 expression had a good response to CTLA4 − /PD1 + , CTLA4 + /PD1 + immunotherapy. The TSPAN4/12/18/23/25/28/29/31 different expression subgroups did not demonstrate significant differences for CTLA/PD1 treatment. Significance was set at p < 0.05.

In addition, we evaluated the association between the differentially expressed TSPANs and immune checkpoints. Immune checkpoint inhibitors are among the most well-studied and widely used tumor immunotherapies. As demonstrated in the heat maps in Fig. 6A, the expression of TSPANs was closely related to numerous clinically relevant immune checkpoint molecules, again illustrating the high theoretical significance of developing novel immunotherapies for GBM through the TSPAN family.

We then downloaded GBM immunotherapy-related data from TCGA and TCIA databases for further analysis to explore whether the predictive value of TSPANs in tumor immunotherapy could be validated in clinical practice. As presented in Fig. 6B, different immunotherapies resulted in significant differences between the low- and high-expression groups of multiple TSPAN genes. For example, the high TSPAN3 subgroup responded well to anti-CTLA4 treatment, the high TSPAN6 subgroup responded better to anti-CTLA4 and combined anti-CTLA4 and PD1 treatments, while the high TSPAN11 subgroup responded poorly to combined anti-CTLA4 and PD1 treatments. Altogether, these results indicate that TSPANs may play an essential role in the malignant progression of GBM by influencing the TME.

### Survival prediction study of TSPANs in patients with glioma

**Figure 7:**
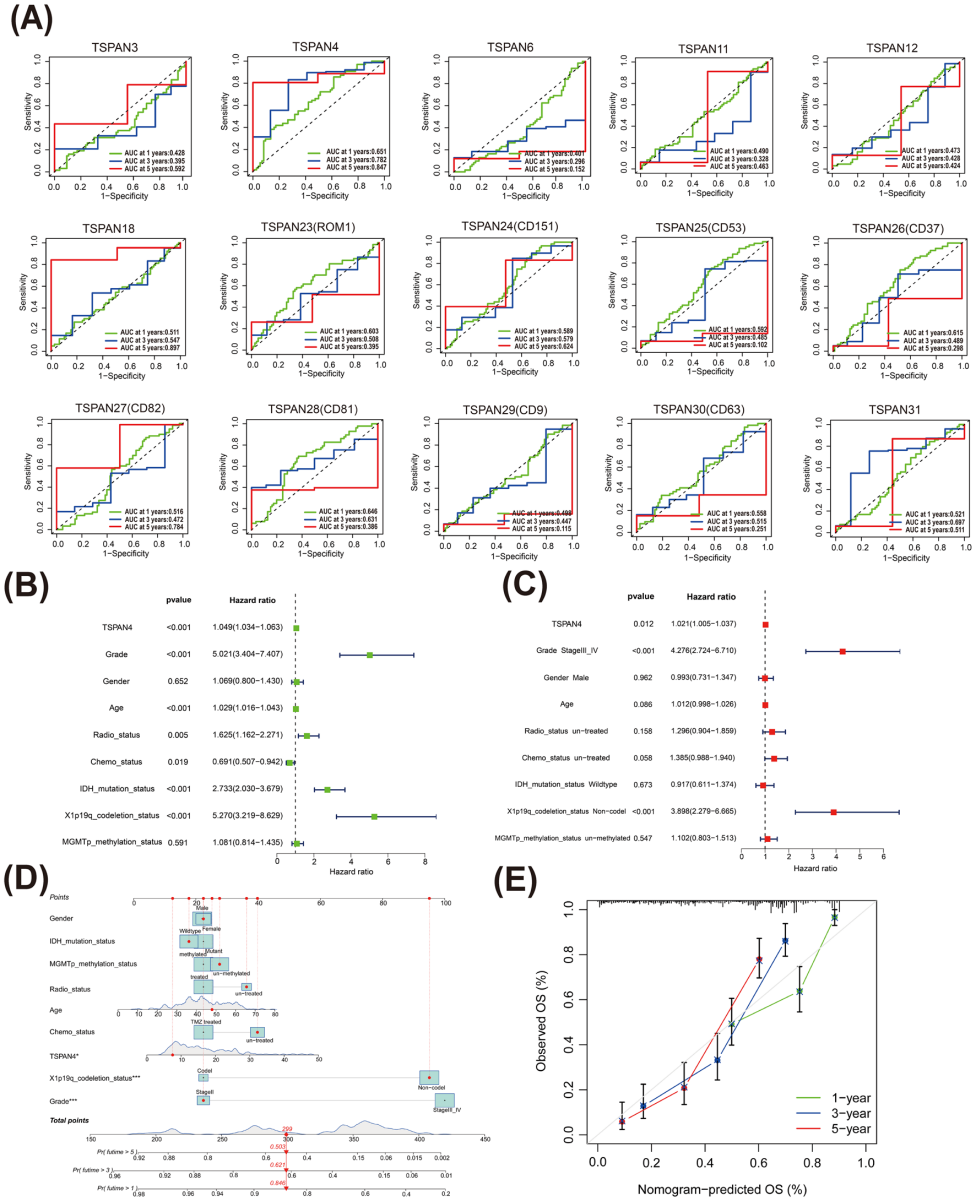
Analysis of the prognostic value of TSPANs. (**A**) Receiver operating characteristic curves of TSPANs based on the TCGA database. Low accuracy: 0.5 < AUC-ROC ≤ 0.7, moderate accuracy: 0.7 < AUC-ROC ≤ 0.9, and high accuracy: 0.9 < AUC-ROC ≤ 1. Univariate Cox analysis (**B**) and multivariate Cox analysis (**C**) were performed to determine whether TSPAN4 was a prospective prognostic indicator and an independent risk factor for glioma. (**D**) A prognostic nomogram was constructed based on the nine variables in the model, including TSPAN4 expression levels; nine corresponding “integral” values can be obtained, which can be summed to calculate the “total score”. Therefore, patients’ 1 -, 3 -, and 5-year overall survival (OS) rates can be predicted. (**E**) Validation of calibration curves for predicting 1 -, 3 -, and 5-year OS. The nomogram’s predicted and actual survival probability were plotted on the X and Y axes, respectively. The diagonal line represents a perfect prediction.

To refine the survival prediction mapping of differentially expressed TSPANs in patients with glioma, we performed an ROC analysis. The AUC of TSPAN4/18/24/27/31 for predicting patient 1-, 3-, and 5-year survival rates was greater than 0.5, which was predictive. Among them, the AUC values of TSPAN4 time-dependent OS were the largest (0.651, 0.782, and 0.847, respectively), proving its good prognostic performance (Fig. 7A).

Next, we used univariate and multivariate Cox regression analyses based on the CGGA dataset to determine whether TSPAN4 expression was an independent risk factor for poor prognosis in patients with glioma. The univariate Cox analysis revealed that TSPAN4 (HR = 1.049; 95% CI  1.034–1.063; p < 0.001), grade (HR = 5.021; 95% CI  3.404–7.407; p < 0.001) , age (HR = 1.029; 95% CI  1.016–1.043; p < 0.001) , radio status(HR = 1.625; 95% CI  1.162–2.271; p < 0.05), chemo status (HR = 0.691; 95% CI  0.507–0.942; p < 0.05), IDH mutation status (HR = 2.733; 95% CI  2.030–3.679; p < 0.001), and X1p19q codeletion status (HR = 5.270; 95% CI  3.219–8.629; p < 0.001) were high-risk factors(Fig. 7B). In the multivariate Cox proportional hazard regression analyses, TSPAN4 was independently associated with overall survival, suggesting that TSPAN4 may play an important role in the malignant process of glioma (HR = 1.021; 95% CI  1.005–1.037; p < 0.05). In addition, two classical clinical risk factors, high grade and X1p19q co-deletion were significantly associated with poorer prognosis in patients with GBM (Fig. 7C).

To more accurately and quantitatively predict the individual survival chances of patients with glioma, we established a quantitative prognostic nomogram model based on risk scores and clinical features, and the same nine variables as COX regression were included, of which grade, X1p19q co-deletion status, and TSPAN4 expression were the first three most influential factors contributing to outcome events (Fig. 7D). Calibration curves were used to estimate the accuracy of the model in predicting the individual outcomes. As presented in Fig. 7E, the calibration curves at 1, 3, and 5 years demonstrated good consistency between the predictions of the nomogram and the actual observed OS, indicating that the nomogram we constructed could accurately predict the future survival of patients with glioma.

### TSPAN4 knockdown inhibits the malignant process of GBM cells

**Figure 8:**
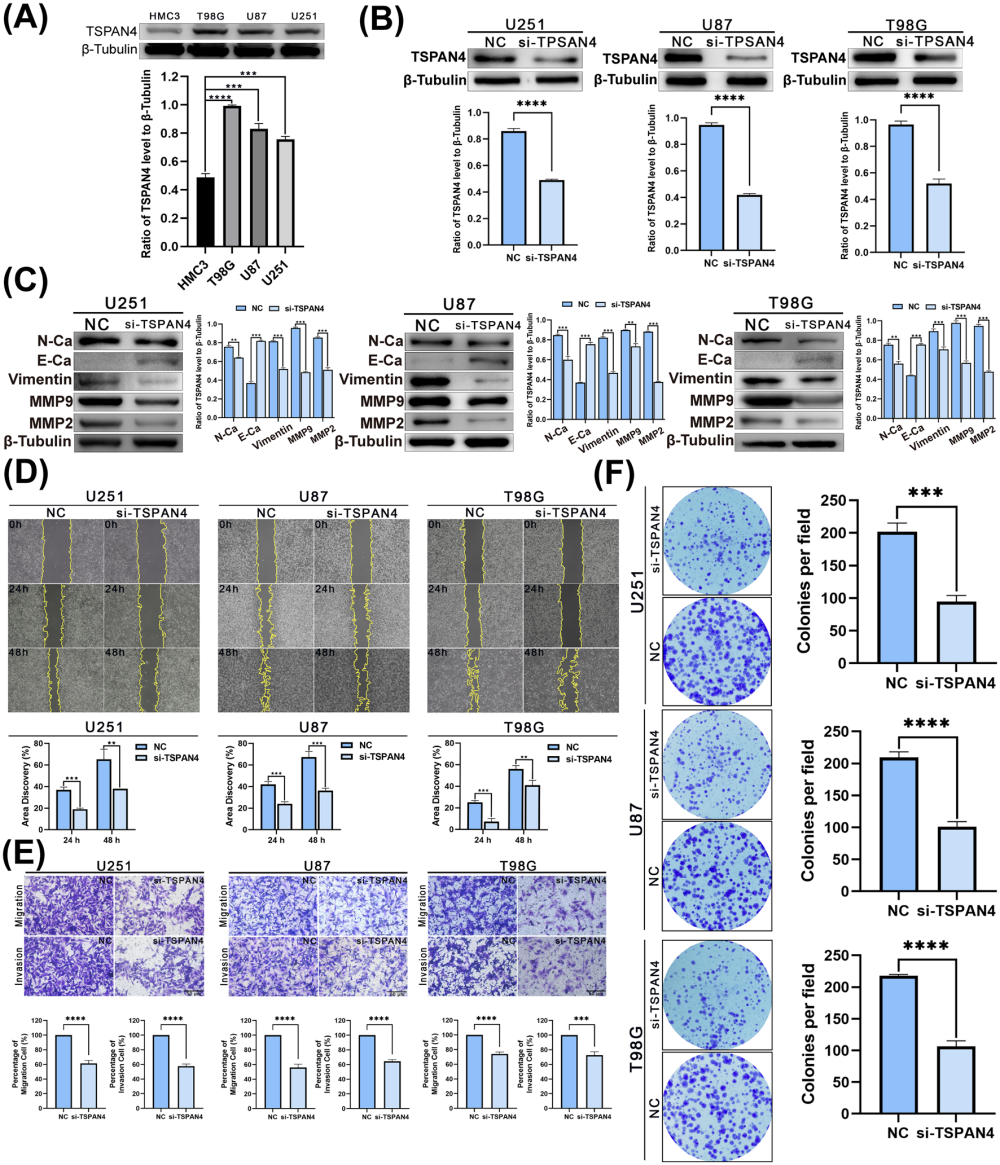
Effect of TSPAN4 expression level on glioblastoma cells. (**A**) Western blotting (WB) results indicated that the expression of TSPAN4 was significantly upregulated in glioma cell lines than in human normal glial cells. (**B**) Using small interfering RNA to transfect glioma cells, the WB results confirmed that TSPAN4 was knocked down. (**C**) The WB results demonstrated that the expression of mesenchymal markers N-cadherin and Vimentin, matrix metalloproteinases MMP2 and MMP9 decreased after TSPAN4 knockdown. The expression of epithelial marker E-cadherin was increased. **p < 0.01, ***p < 0.001, ****p < 0.0001. (**D**) The results of wound healing assays revealed increased healing area after the TSPAN4 knockdown. (**E**) A Transwell assay was performed to detect migration and invasion images in the NC group and TSPAN4 knockout group and to quantify the analysis. (**F**) The colony assay revealed that TSPAN4 knockdown resulted in smaller colony size and reduced colony number. **p < 0.01, ***p < 0.001, ****p < 0.0001.

Considering that TSPAN4 is highly expressed in GBM and closely associated with poor prognosis, TSPAN4 may likely be an oncogene in GBM. Three GBM cell lines, U251, U87, and T98G, were used for the in vitro experiments to test this hypothesis. qRT-PCR revealed that TSPAN4 was expressed in all the three cell lines (Additional File 5). Western blotting confirmed that the expression of TSPAN4 was significantly higher in glioma cell lines than in normal human glial cells HMC3 (Fig. 8A). Cell lines were transfected with small interfering RNA (siRNA) to explore the effect of specific silencing of TSPAN4 expression on GBM cell proliferation, invasion, and migration. Western blotting was used to assess the efficacy of transfection, and the results indicated successful knockdown (Fig. 8B).

Our results revealed that the expression of the mesenchymal markers N-cadherin, Vimentin, and matrix metalloproteinase 2/9 (MMP2/MMP9) was decreased after TSPAN4 knockdown compared to the control in the three cell lines. By contrast, expression of the epithelial marker E-cadherin increased (Fig. 8C). These results indicated that TSPAN4 is associated with the expression of EMT marker molecules that predict the malignant process of tumors.

After 24 h and 48 h of the wound-healing assay, trauma spacing was significantly more expansive, and trauma healing was slower in the TSPAN4 knockdown group than in the control group (Fig. 8D). The results of the Transwell assay revealed that the invasion and migration abilities of the three cell lines were significantly decreased, and invasion and metastasis were blocked (Fig. 8E). Colony formation assays were performed to determine the effect of TSPAN4 expression on GBM cell proliferation. The experimental results are presented in Fig. 8F. Compared to the control group, the number of colonies formed in GBM cells was significantly reduced after the down-regulation of TSPAN4. Therefore, TSPAN4 plays an essential role in the malignant process of GBM proliferation, invasion, and migration and would be a promising molecular therapeutic target.

## Discussion

Glioblastoma remains an incurable disease with strong invasiveness, and its progressive infiltration of the surrounding brain parenchyma can dramatically affect the patients' cognitive function and quality of life. Most existing treatments are palliative, with few survival benefits^23^. Therefore, identifying novel molecular targets with promising applications is crucial. Because tetraspanins have been demonstrated to regulate metastasis and malignant progression in various tumors and participate in immune response mechanisms, they have high potential for practical applications. Anti-TSPAN26 antibody have been used in clinical trials to treat relapsed/refractory chronic lymphocytic leukemia^24^.

However, the molecular function of TSPANs remains hypothetical. First, owing to a large number of TSPAN family members, studies on their complete structure remain scarce^7^. Second, TSPANs as signaling molecules are widely and abundantly expressed in cell membranes, making their functional contributions challenging to classify^25^. Previous studies have focused on one or a few TSPAN proteins, uncovering many unrelated processes that are dependent on tetraspanins. However, information on the other family members is limited, and leading scientists attempt to clarify the general functional principles of tetraspanins. To the best of our knowledge, this is the first study to systematically elucidate the expression profile, functional enrichment, immune infiltration, and prognostic value of the TSPAN family in GBM using a bioinformatics approach, in which 15 differentially expressed members were analyzed without discrimination. In particular, we extended the study of previously neglected TSPANs and performed an in vitro experimental validation of TSPAN4, which has the most significant predictive prognostic value. We believe that our efforts contribute to the future understanding of the generic functions of tetraspanin proteins in GBM and screen pivotal members to provide a platform for developing new targeted/immunotherapy therapies.

TSPAN4 is a potential biomarker of hepatocellular carcinoma and plays a crucial role in promoting cancer cell proliferation^26^; however, its role in GBM remains largely unexplored. In this study, analysis of the GEPIA database revealed that the mRNA level of TSPAN4 was significantly upregulated in GBM compared to normal tissues. Analysis of the CGGA online database demonstrated that higher levels of TSPAN4 were associated with a poorer prognosis in patients with GBM. In addition, TSPAN4 expression was significantly correlated with GBM patient age, tumor grade, IDH mutation status, 1p/19q co-deletion status, and drug resistance. The increased immune and stromal scores in the TSPAN4 high-expression group may provide new ideas for investigating the dynamic regulation of immune and stromal components of the GBM TME and deciphering the TME-related gene signature. Bioprocess enrichment analysis revealed that TSPAN4 is mainly associated with humoral immune response, leukocyte migration, and immunoglobulin superfamily domain construction, reaffirming that it may be a key factor affecting immunotherapy in GBM. The AUC values of TSPAN4 for predicting the 1-, 3-, and 5-year survival rates of patients were all > 0.6, suggesting good prognostic performance. The univariate and multivariate analyse revealed that TSPAN4 is an independent risk factor for poor prognosis in patients with GBM. Data from the GSCA platform indicated that TSPAN4 significantly activates the EMT pathway. Further in vitro experiments also demonstrated that TSPAN4 knockdown was followed by the downregulation of EMT marker protein expression and reduced proliferation, invasion, and migration of glioma cells. Recent studies have reported that TSPAN4 is abundant in the migrasome as a marker protein, and can effectively promote migrasome formation. The migrasome is an organelle that may arise from immune and tumor cells during metastasis. They integrate spatial, temporal, and specific chemical information to mediate intercellular communication, which is closely related to cell migration function^27^. Our GSEA results indicated that TSPAN4 is closely related to the chemokine signaling pathway and cytokine–cytokine receptor interactions, while migrasome are enriched for chemokines and cytokines. Thus, the high expression of TSPAN4 in GBM may play a vital role in the malignant progression of glioma cells and the formation of the TME, and that the migrasome is a promising mechanism. Taken together, TSPAN4 may be a promising biomarker for designing novel therapies and improving the accuracy of prognostic predictions in patients with GBM.

TSPAN6/18/24/25/26/29/30 were significantly highly expressed in GBM in our analysis, and all predicted poor prognosis. They are also associated with clinical features of gliomas to varying degrees. Previous studies have confirmed that TSPAN6 regulates colorectal cancer carcinogenesis^28^; however, its role in GBM remains unknown. Our analysis revealed that the high TSPAN6 subgroup responded well to anti-CTLA4 and a combination of anti-CTLA4 and PD1 therapy. Further clinical data are required to validate the exact effects of TSPAN6. TSPAN18 has been neglected in previous tumor studies. Our results revealed that TSPAN18 was mainly enriched in toll-like receptor signaling pathways, natural killer cell-mediated cytotoxicity, and Rig-i-like receptor signaling pathway, demonstrating its indispensable role in the immune response. Meanwhile, TSPAN18 has good predictive performance for the 1-, 3-, and 5-year survival rates of patients with glioma.

TSPAN24 (CD151) interacts with integrins to enhance adhesion^29^ and is involved in pathological angiogenesis^30^. CD151-specific monoclonal antibodies can strongly inhibit the growth and metastasis of primary tumors^31^. Our results suggest that TSPAN24 is a promising target because it is associated with resistance to various small-molecule-targeted antitumor drugs and can be a good prognostic predictor in patients with glioma.

TSPAN25(CD53) and TSPAN26(CD37), which are specifically expressed in the immune system^32^ and interact with MHC II^33,34^, have been identified in exosomes from B cells^35^. Our data revealed a significantly higher immune/stromal score for highly expressed TSPAN25/26 and a positive correlation with M2-type macrophages. Previous studies have confirmed that the predominantly infiltrating tumor-associated macrophages (TAMs) in gliomas are immunosuppressive M2 macrophages, which not only lead to the formation of an immunosuppressive TME but also promote the malignant progression of gliomas. A high proportion of M2 cells predicts a higher grade and malignancy, as well as lower long-term survival^36,37^. The GSEA enrichment analysis revealed that TSPAN25/26 is closely associated with immunoglobulin production, cellular immunity, humoral immunity, and antigen-binding processes, further supporting its possible role in the GBM immune microenvironment.

TSPAN29 (CD9) and TSPAN30 (CD63) are widely expressed in the immune system^32,38^, such as a study reporting that CD63 co-localizes with cytotoxic granules in eosinophils^39^. Our results revealed that TSPAN30 was significantly negatively associated with eosinophils, although the underlying mechanisms remain to be elucidated. Interestingly, TSPAN30 was the only tetraspanin that was significantly associated with all six clinical characteristics: grading, IDH mutation status, 1p/19q co-deletion status, sex, age, and recurrence, suggesting that more research is needed regarding the role of TSPAN30 in GBM. The results for CD9 are contradictory because CD9 is generally a suppressor of tumor invasion and metastasis; however, in specific situations, CD9 may play a role in promoting invasion^25,40,41^. The results of a previous study from *Drosophila* to human glioma suggested an apparently negative role for CD9 in glioma^42^, which is consistent with our results. CD9 and CD63 also interact with MHC II and regulate its function^34,43^. Our results revealed that immune/stroma scores were significantly higher in both TSPAN29/30 high expression groups, suggesting that they may affect the tumor immune microenvironment in GBM by regulating pattern recognition, antigen presentation, and T-cell activation (TSPAN29 positively correlated with Tfh and negatively correlated with CD4 + memory T cells).

TSPAN12/23/27/28/31 did not reveal a clear prognostic value in CGGA survival analysis. However, other analyses yielded many positive results. Among them, TSPAN12 was significantly decreased in patients with WHO grade IV, IDH wild type, and age ≥ 42 years old, and its immune/stroma score was also significantly lower in the high expression group. We reasonably suspect that TSPAN12 may play a positive role in gliomas. However, the exact underlying mechanism requires further investigation. TSPAN23/27/28/31 demonstrated significance in the ROC curve analysis, indicating that they may also play a role in the occurrence and development of gliomas.

Unlike the abovementioned 13 TSPAN proteins, the high expression of TSPAN3 and TSPAN11 predicted a better prognosis. In the subsequent analysis, we further found that the levels of TSPAN3/11 were reduced in WHO IV with statistically significant differences. Moreover, in the clinical correlation analysis, TSPAN3/11 expression was significantly reduced in patients with wild-type IDH, 1p/19q non-codel, recurrent tumors, and age > 42 years. We also found higher stromal immune scores for low TSPAN3/11, with statistically significant differences. However, studies on the role of TSPAN3/11 in glioma are lacking. We hope that our results will help clarify whether these compounds inhibit glioma progression.

## Conclusions

To the best of our knowledge, this study is the first systematic investigation of the expression profiles and functions of the tetraspanin family in gliomas, filling a gap in previous glioma studies on most family members. Additionally, we selected TSPAN4 with the best prognostic performance for experimental validation and demonstrated that the proliferation, invasion, and migration of glioma cells were significantly inhibited after TSPAN4 knockdown. However, this study has some limitations. First, each of the tetraspanin family members was not verified owing to their large number. Second, although a detailed analysis of each differentially expressed tetraspanin was performed, and an attempt was made to link them together based on common results, further in-depth studies on their connections and interactions are needed. We hope that our results can serve as an excellent starting point to encourage further more discussions and reflections on the mechanism of tetraspanins in glioma and provide a theoretical basis for discovering new prognostic biomarkers and immune-related therapeutic targets for glioma.

### Supplementary Information

Supplementary Information 1.

Supplementary Information 2.

Supplementary Information 3.

Supplementary Information 4.

Supplementary Information 5.

Supplementary Information 6.

## Data Availability

Original datasets are available in a publicly accessible repository: Gene Expression Profiling Interactive Analysis (GEPIA) (http://gepia.cancer-pku.cn/). The Chinese Glioma Genome Atlas (CGGA) database (http://www.cgga.org.cn). The Human Protein Atlas (HPA) (https://www.proteinatlas.org/). The Cancer Genome Atlas (TCGA) database (https://portal.gdc.cancer.gov/). The Cancer Imaging Archive (TCIA) (https://cancerimagingarchive.net/). FunRich (http://www.funrich.org). DAVID Knowledgebase v2022q3 (https://david.ncifcrf.gov/). The cBioPortal platform (http://www.cbioportal.org/). The Gene Set Cancer Analysis (GSCA) database (http://bioinfo.life.hust.edu.cn/GSCA/).

## Abbreviations

GBM: Glioblastoma
TSPANs: Tetraspanins
GSEA: Gene set enrichment analysis
GO: Gene ontology
KEGG: Kyoto encyclopedia of genes and genomes
TME: Tumor microenvironment
TM4SF: Transmembrane 4 superfamily
TMs: Transmembrane domains
TEMs or TERMs: Tspan-enriched microstructural domains
GEPIA: Gene expression profiling interactive analysis
CGGA: The Chinese glioma genome atlas
HPA: The human protein atlas
TCGA: The cancer genome atlas
TCIA: The cancer imaging archive
GSCA: The gene set cancer analysis
FDA: The food and drug administration
FBS: Foetal bovine serum
PRAD: Prostate adenocarcinoma
READ: Rectum adenocarcinoma
SKCM: Skin cutaneous melanoma
TGCT: Testicular germ cell tumors
CHOL: Cholangio carcinoma
COAD: Colon adenocarcinoma
LGG: Brain lower grade glioma
PAAD: Pancreatic adenocarcinoma
OS: Overall survival
IDH1: Isocitrate dehydrogenase 1
IDH2: Isocitrate dehydrogenase 2
WHO: The World Health Organization
Tfh: Follicular helper T cells
AUC: Area under the curve
ROC: Receiver operating characteristic
siRNA: Small interfering RNA
MMP2/MMP9: Matrix metalloproteinases 2/9
TAMs: Tumor-associated macrophages
