# Supplementary material for: Tetraspanins predict the prognosis and characterize the tumor immune microenvironment of glioblastoma
Source: Sci Rep. 2023 Aug 16;13:13317. doi: 10.1038/s41598-023-40425-w (PMC10432458; doi:10.1038/s41598-023-40425-w)
Supplement: Supplementary file 1 — Supplementary Information 1. [file 41598_2023_40425_MOESM1_ESM.pdf]

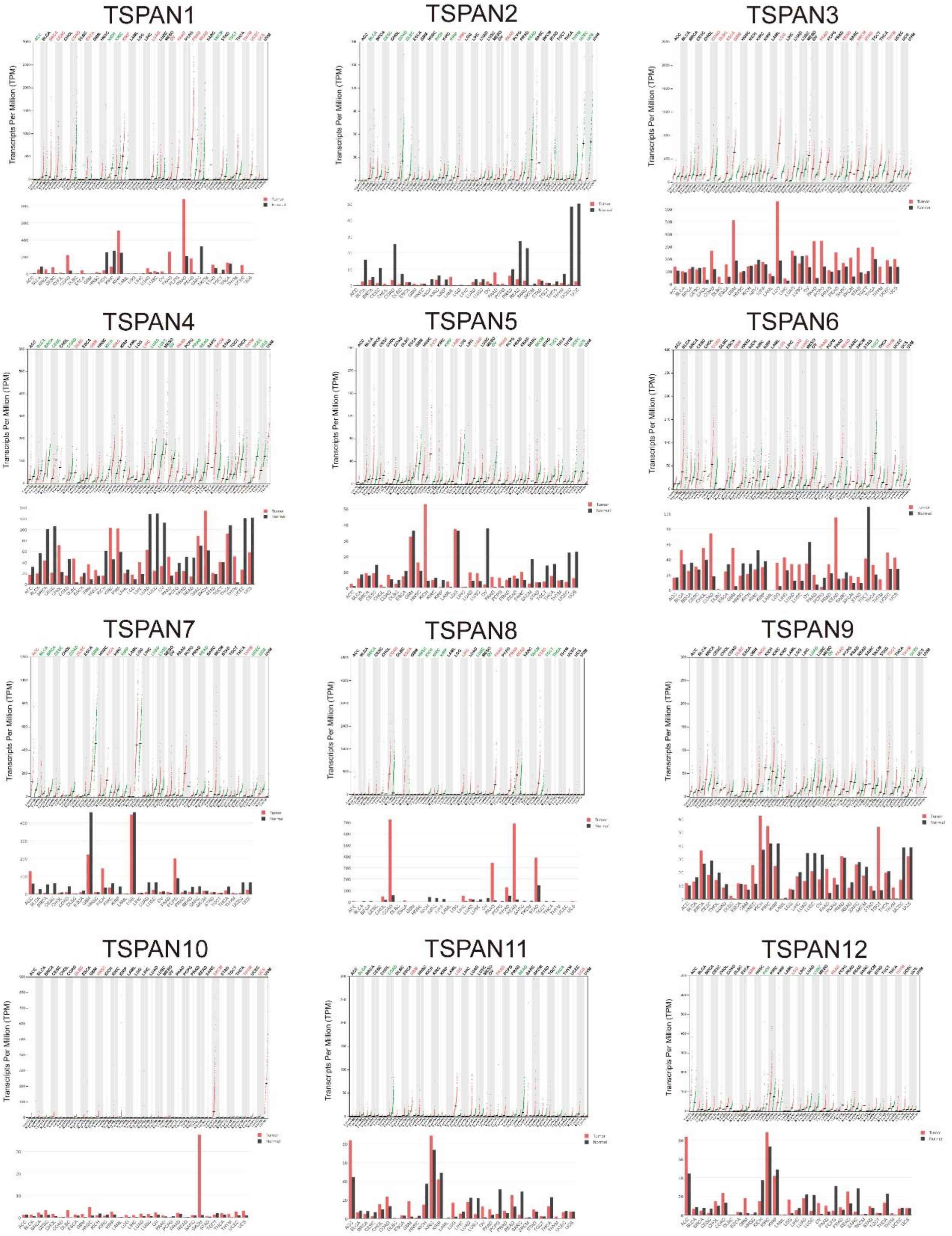
TSPAN1
TSPAN2
TSPAN3
TSPAN4
TSPAN5
TSPAN6
TSPAN7
TSPAN8
TSPAN9
TSPAN10
TSPAN11
TSPAN12
Transcripts Per Million (TPM)

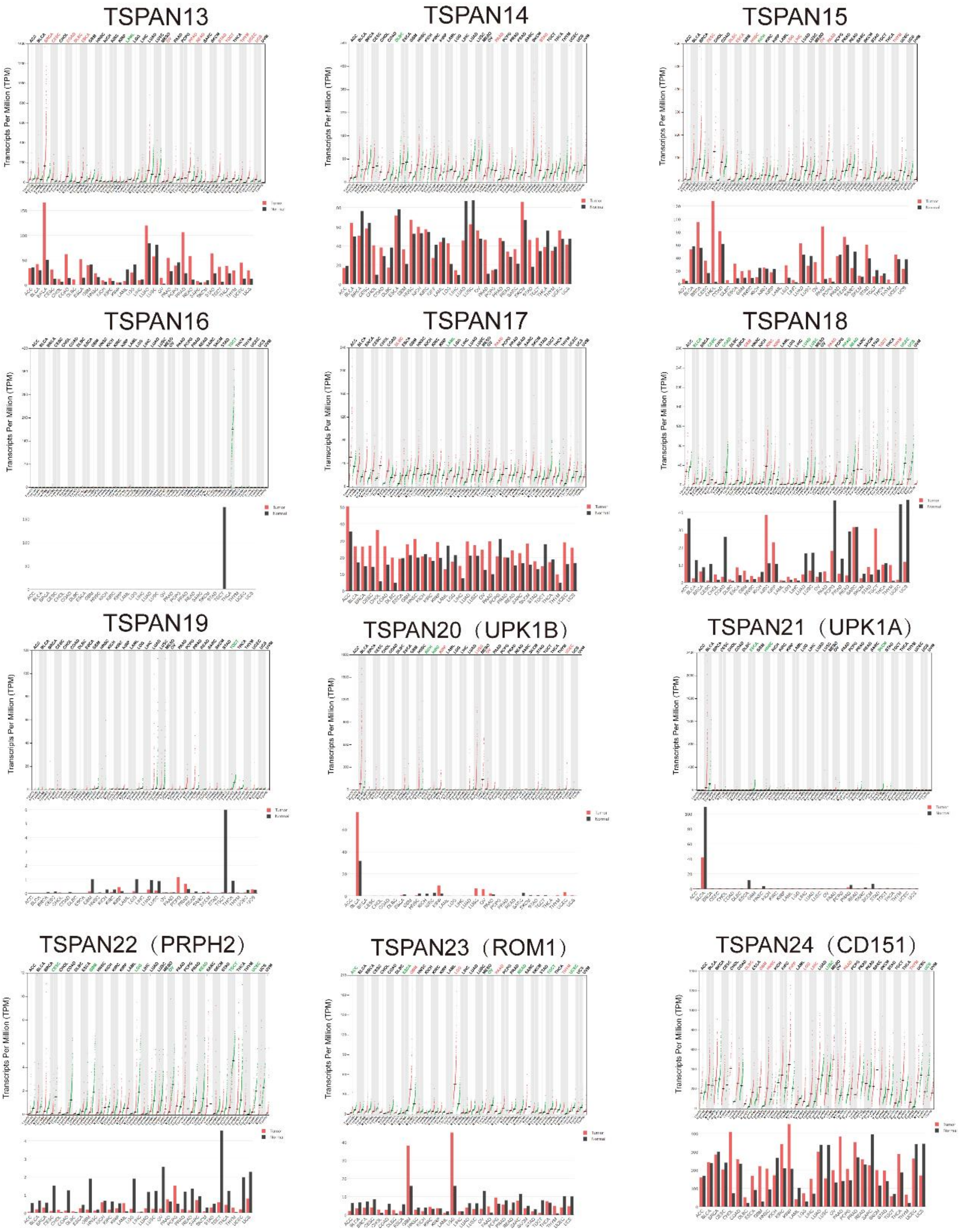
TSPAN13
TSPAN14
TSPAN15
TSPAN16
TSPAN17
TSPAN18
TSPAN19
TSPAN20（UPK1B）
TSPAN21（UPK1A）
TSPAN22（PRPH2）
TSPAN23（ROM1）
TSPAN24（CD151）
Transcripts Per Million (TPM)

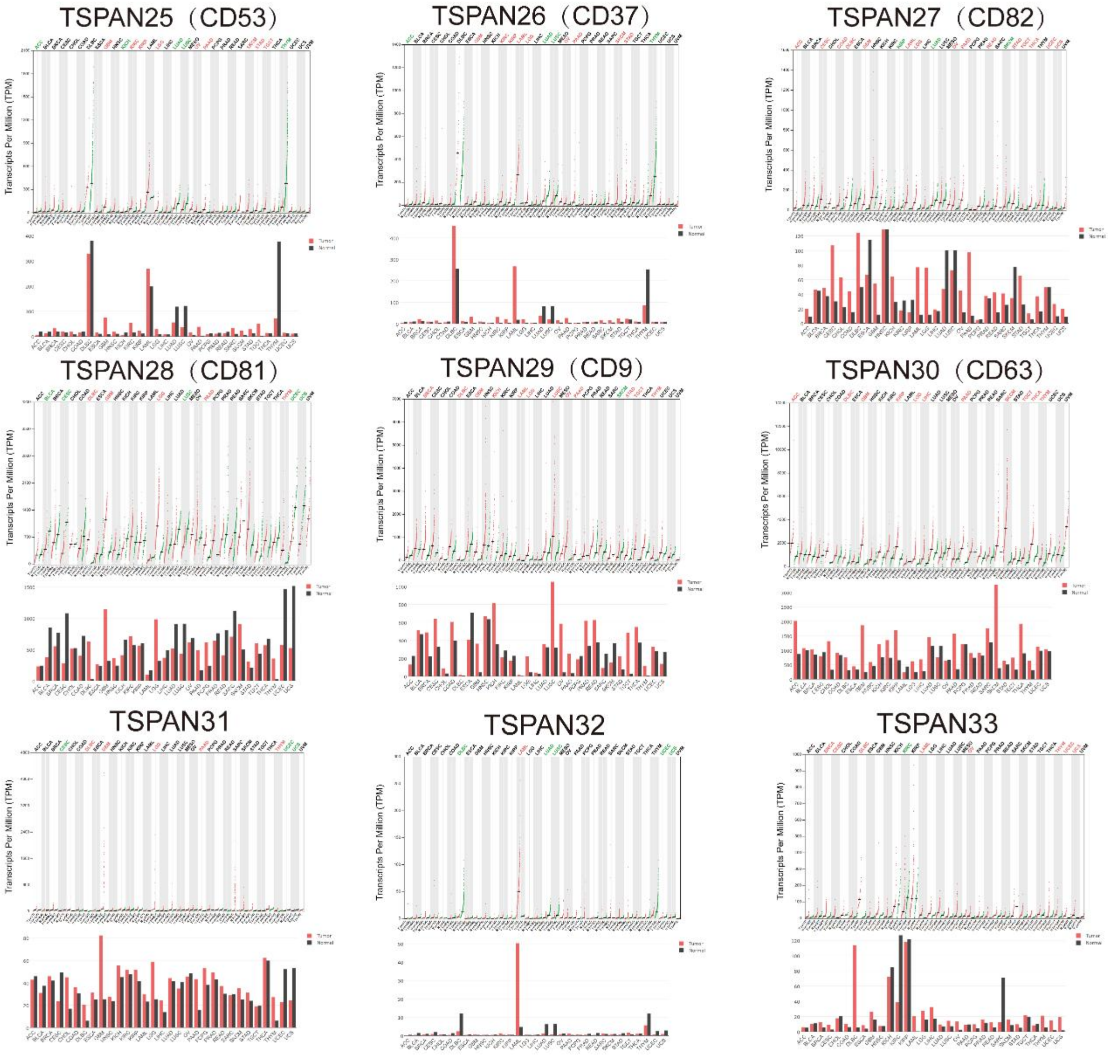

Additional file 1 Analysis of pan-cancer TSPAN expression levels using the Gene Expression Profiling Interactive Analysis database.
