# Supplementary material for: Tetraspanins predict the prognosis and characterize the tumor immune microenvironment of glioblastoma
Source: Sci Rep. 2023 Aug 16;13:13317. doi: 10.1038/s41598-023-40425-w (PMC10432458; doi:10.1038/s41598-023-40425-w)
Supplement: Supplementary file 3 — Supplementary Information 3. [file 41598_2023_40425_MOESM3_ESM.pdf]

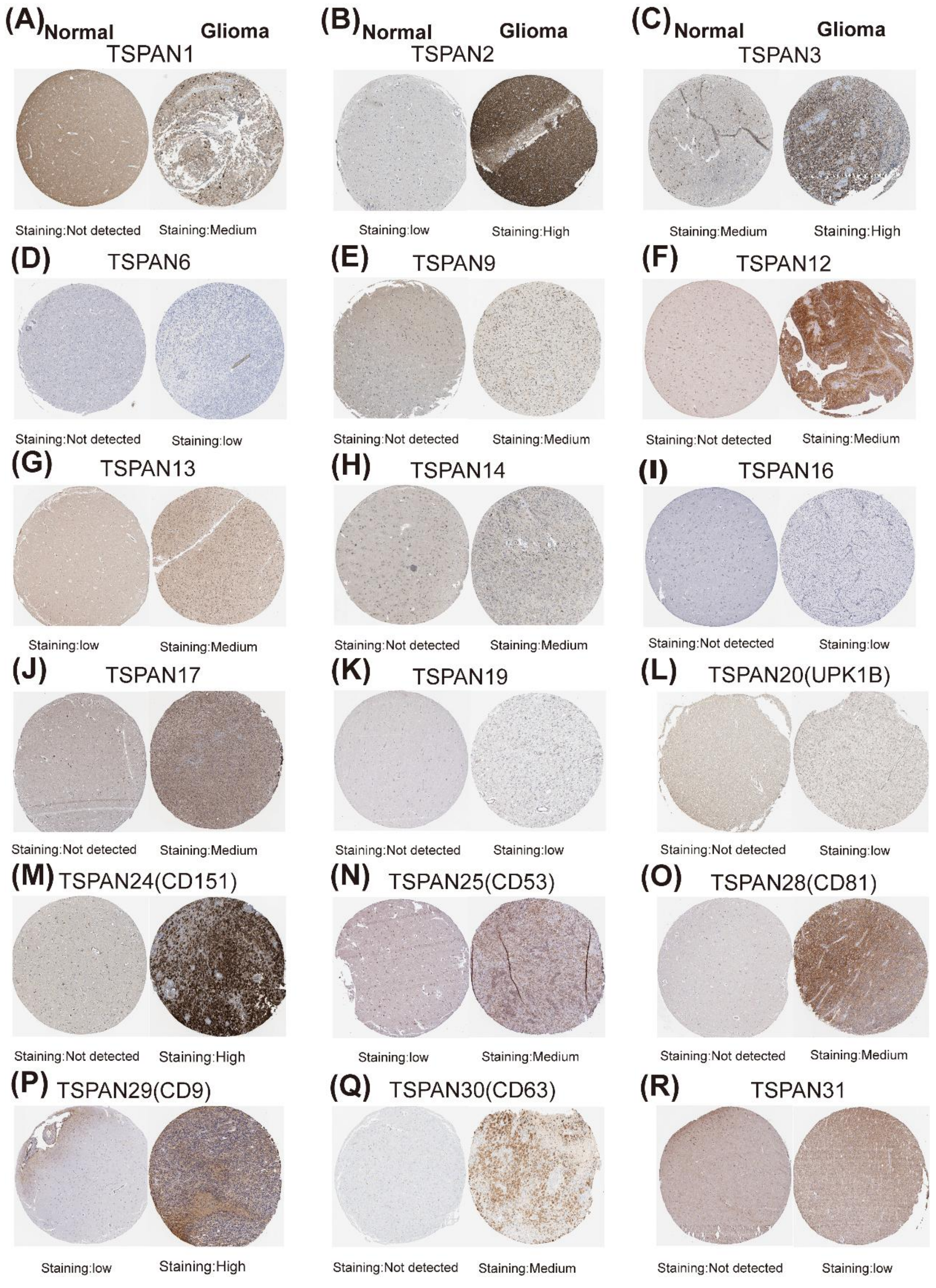

Additional file 3 Protein expression of TSPANs in normal and glioma tissues. Immunohistochemical data were obtained from Human Protein Atlas.
