# Supplementary material for: Tetraspanins predict the prognosis and characterize the tumor immune microenvironment of glioblastoma
Source: Sci Rep. 2023 Aug 16;13:13317. doi: 10.1038/s41598-023-40425-w (PMC10432458; doi:10.1038/s41598-023-40425-w)
Supplement: Supplementary file 4 — Supplementary Information 4. [file 41598_2023_40425_MOESM4_ESM.pdf]

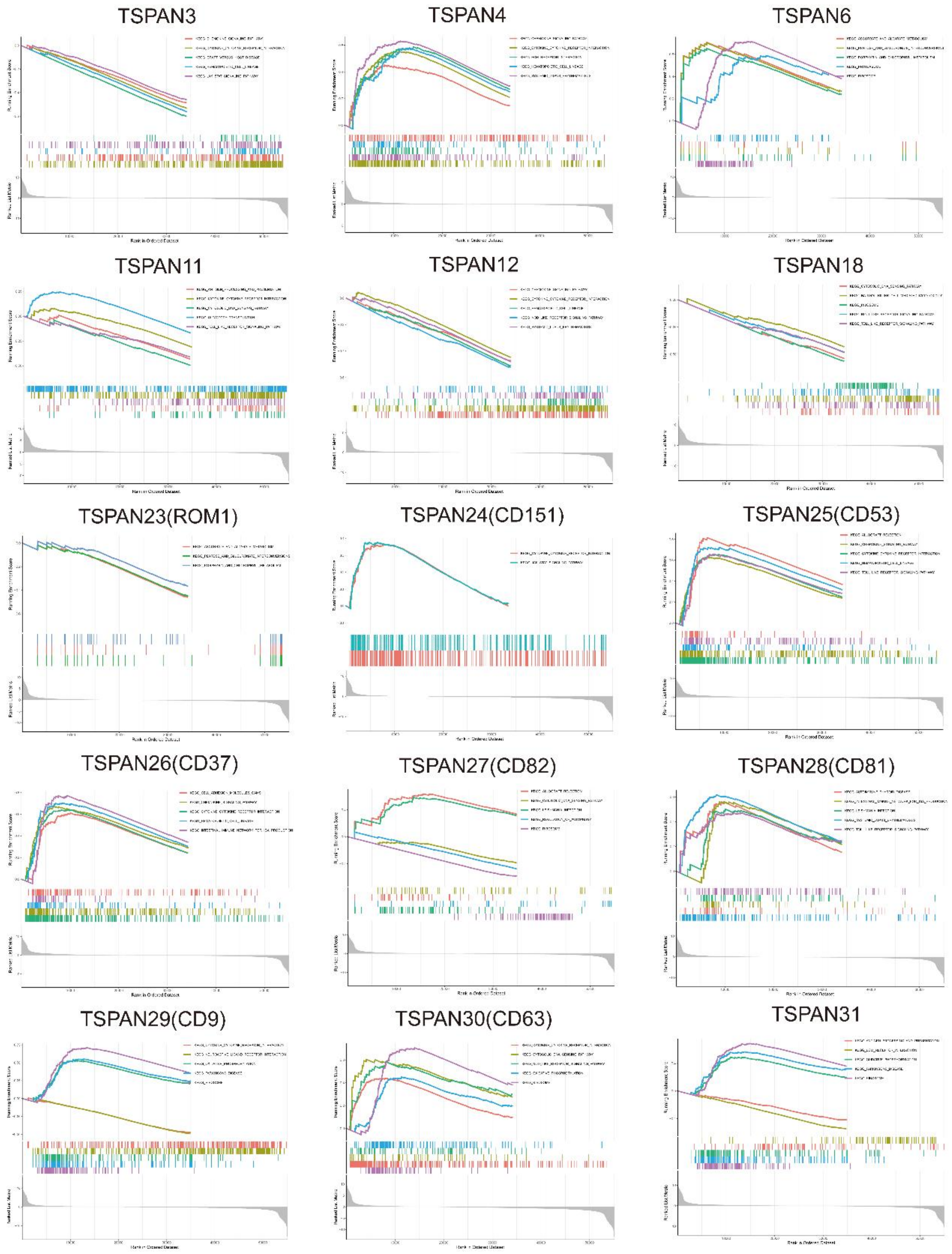

Additional file 4 Gene set enrichment analysis revealed that the differentially expressed TSPANs were mainly enriched in cytokine-cytokine receptor interactions, antigen processing, and presentation.
