# Supplementary figures and images for: Tetraspanins predict the prognosis and characterize the tumor immune microenvironment of glioblastoma
Source: Sci Rep. 2023 Aug 16;13:13317. doi: 10.1038/s41598-023-40425-w (PMC10432458; doi:10.1038/s41598-023-40425-w)

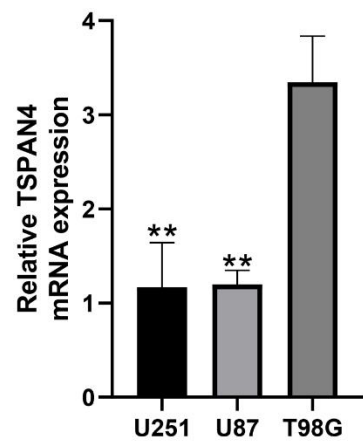

**Additional file 5** qRT-PCR confirmed that TSPAN4 was expressed in all three glioma cell lines. \*\* $p < 0.01$ .

Supplement: Supplementary file 5 — Supplementary Information 5. [file 41598_2023_40425_MOESM5_ESM.pdf]

TSPAN4-26kDa

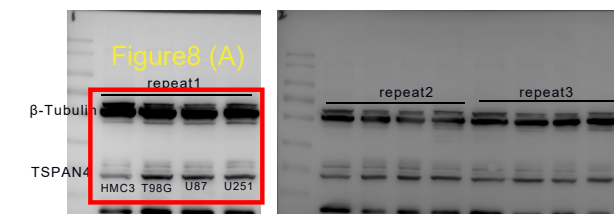

T98G si-TSPAN4-26kDa

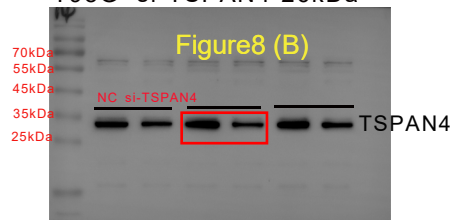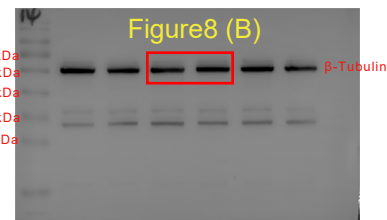

U251 si-TSPAN4-26kDa

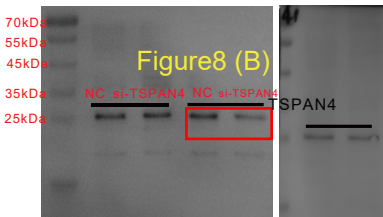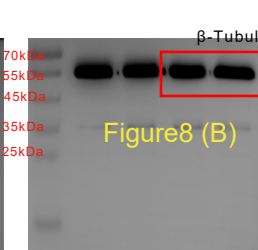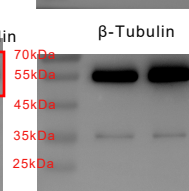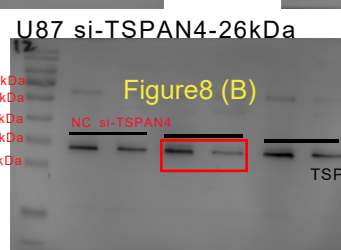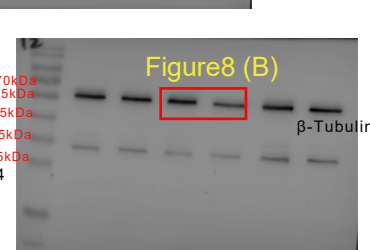

U251 MMP2-62kDa

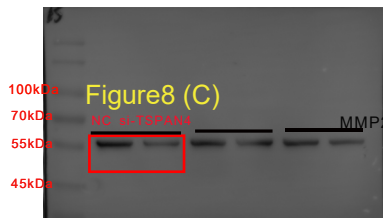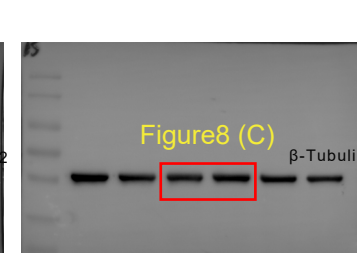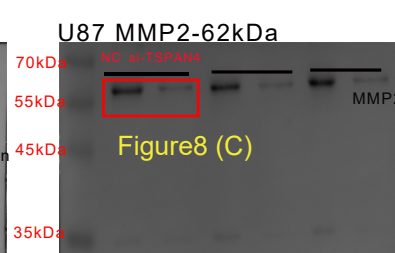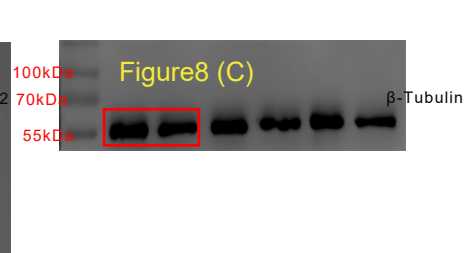

T98G MMP2-62kDa

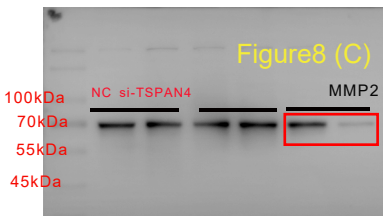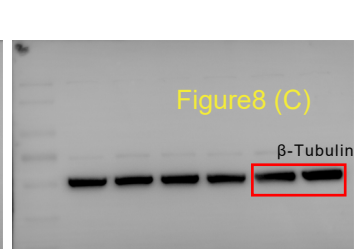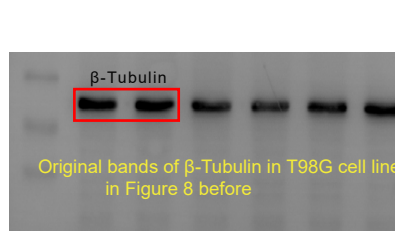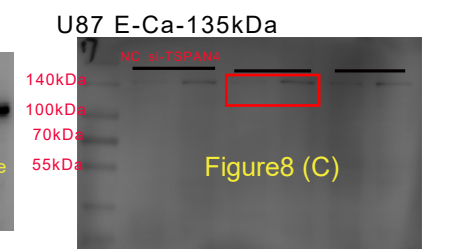

U251 E-Ca-135kDa

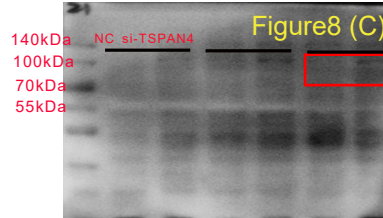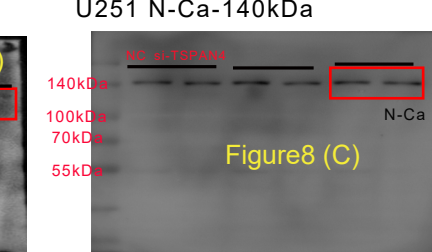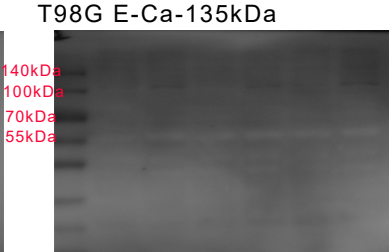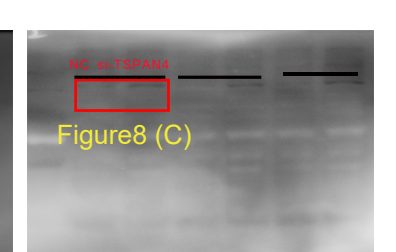

U87 N-Ca-140kDa

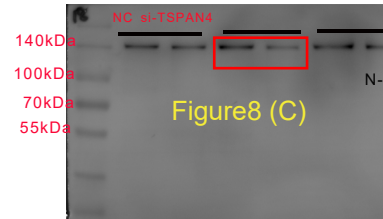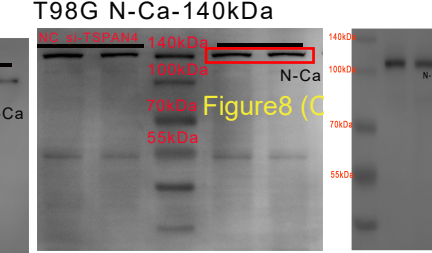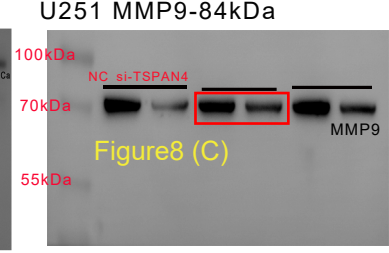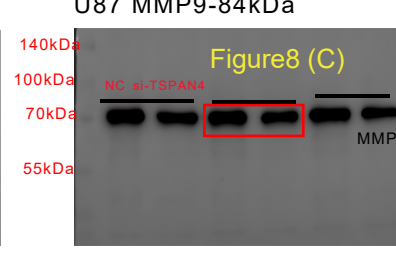

T98G MMP9-84kDa

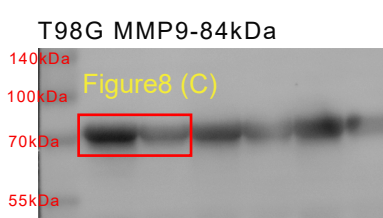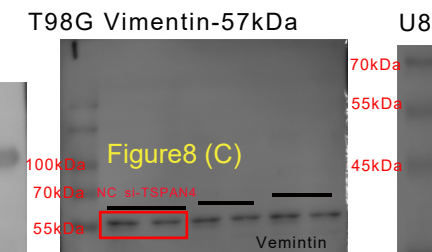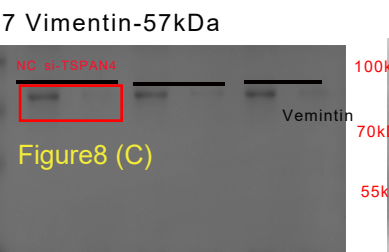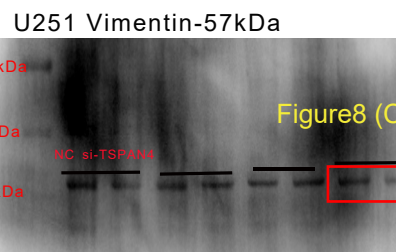

Supplement: Supplementary file 6 — Supplementary Information 6. [file 41598_2023_40425_MOESM6_ESM.pdf]
